# Linear space string correction algorithm using the Damerau-Levenshtein distance

**DOI:** 10.1186/s12859-019-3184-8

**Published:** 2020-12-09

**Authors:** Chunchun Zhao, Sartaj Sahni

**Affiliations:** Department of Computer and Information Science and Engineering, University of Florida, Gainesville, 32611 FL USA

**Keywords:** Edit distance, Damerau-Levenshtein distance, Linear space, String correction

## Abstract

**Background:**

The Damerau-Levenshtein (DL) distance metric has been widely used in the biological science. It tries to identify the similar region of DNA,RNA and protein sequences by transforming one sequence to the another using the substitution, insertion, deletion and transposition operations. Lowrance and Wagner have developed an *O*(*mn*) time *O*(*mn*) space algorithm to find the minimum cost edit sequence between strings of length *m* and *n*, respectively. In our previous research, we have developed algorithms that run in *O*(*mn*) time using only *O*(*s*∗min{*m,n*}+*m*+*n*) space, where *s* is the size of the alphabet comprising the strings, to compute the DL distance as well as the corresponding edit sequence. These are so far the fastest and most space efficient algorithms. In this paper, we focus on the development of algorithms whose asymptotic space complexity is linear.

**Results:**

We develop linear space algorithms to compute the Damerau-Levenshtein (DL) distance between two strings and determine the optimal trace (corresponding edit operations.)Extensive experiments conducted on three computational platforms–Xeon E5 2603, I7-x980 and Xeon E5 2695–show that, our algorithms, in addition to using less space, are much faster than earlier algorithms.

**Conclusion:**

Besides using less space than the previously known algorithms,significant run-time improvement was seen for our new algorithms on all three of our experimental platforms. On all platforms, our linear-space cache-efficient algorithms reduced run time by as much as 56.4% and 57.4% in respect to compute the DL distance and an optimal edit sequences compared to previous algorithms. Our multi-core algorithms reduced the run time by up to 59.3*%* compared to the best previously known multi-core algorithms.

## Background

### Introduction

The Damerau-Levenshtein (DL) distance between two strings is the minimum number of substitutions, inserts, deletes, and transpositions of adjacent characters required to transform one string into the other. Some of the applications of the DL are spelling error correction [1–3], comparing packet traces [4], data mining and clustering [5], quantifying the similarity of biological sequences, and gene function prediction [6], analysis of B cell receptor repertoire data [7], virus detection in software [8], clustering of RNA-seq read se6gments [9], DNA repeats detection [10], and codes for DNA based storage [11]. In some of these applications (e.g., spelling error correction), the strings are rather small while in others (e.g., comparing protein sequences) the strings could be tens of thousands of characters long [12], and in yet others (e.g., comparing chromosomes) they could be millions of characters long [13].

Other string edit distances used in the literature permit only a proper subset of the operations permitted by the DL distance. For example, in the Levenshtein distance [14] transpositions are not permitted, in the Hamming distance [15] only substitutions are permitted, and in the Jaro distance [16], only transpositions are permitted. The correct distance metric to use depends on the application. In the applications cited above, the DL distance is used as all 4 edit operations are permitted.

Lowrance and Wagner [17] considered a generalization of DL distance to the case when substitutions, inserts, deletes, and transpositions have different costs. Through a suitable choice of weights, the weighted DL distance can be made equal to the DL distance, Levenshtein distance, Hamming distance, and Jaro distance.

Lowrance and Wagner [17] have developed an *O*(*mn*) time and *O*(*mn*) space algorithm to find the minimum cost edit sequence (ie., sequence of substitutions, inserts, deletes, and transpositions) that transforms a given string of length *m* into a given string of length *n* provided that 2*T*≥*I*+*D*, where *T*, *I*, and *D*, are respectively, the cost of a transposition, insertion, and deletion. In the DL distance, *T*=*I*=*D*=1 and so, 2*T*=*I*+*D*. Hence, the algorithm of Lowrance and Wagner [17] may be used to compute the DL distance as well as the corresponding edit sequence in *O*(*mn*) time and *O*(*mn*) space. This observation has also been made in [2]. In [18] we developed algorithms that run in *O*(*mn*) time using only *O*(*s*∗ min{*m,n*}+*m*+*n*) space, where *s* is the size of the alphabet comprising the strings, to compute the DL distance as well as the corresponding edit sequence. Since *s*<<*m* and *s*<<*n* in most applications (e.g., *s*=20 for protein sequences), this reduction in space enables the solution of much larger instances than is possible using the algorithm of [17]. Our algorithms in [18] are much faster as well. In this paper, we develop algorithms to compute the DL distance and corresponding edit sequence using *O*(*m*+*n*) space and *O*(*mn*) time. Extensive experimentation using 3 different platforms indicates that the algorithms of this paper are also faster than those of [18]. In fact, our fastest algorithm for the DL distance is up to 56.4% faster than the fastest algorithm in [18] when run on a single core. The single core speedup to find the corresponding edit sequence is up to 57.4%. Our algorithms may be adapted to run on multicores providing a speedup of up to 59.3%.

### DL dynamic programming recurrences

Let *A*[1:*m*]=*a*_1_*a*_2_⋯*a*_*m*_ and *B*[1:*n*]=*b*_1_*b*_2_...*b*_*n*_ be two strings of length *m* and *n*, respectively. Let *H*_*ij*_ be the DL distance between *A*[1:*i*] and *B*[1:*j*]. So, *H*_*mn*_ is the DL distance between *A* and *B*. The dynamic programming recurrence for *H* is given below [17, 18].

1 $$ H_{i,0} = i,\ H_{0,j} = j, \ 0 \le i \le m, \ 0 \le j \le n  $$

When *i*>0 and *j*>0,

2 $$ H_{i,j} = \min\left\{ \begin{array} {lcr} H_{i-1,j-1}+ c(a_{i},b_{j}) \\ H_{i,j-1}+ 1 \\ H_{i-1,j}+ 1 \\ H_{k-1,l-1} + (i-k-1)+ 1 + (j-l-1) \\ \end{array} \right.   $$

where *c*(*a*_*i*_,*b*_*j*_) is 1 if *a*_*i*_≠*b*_*j*_ and 0 otherwise, *k*=*lastA*[*i*][*b*_*j*_] is the last (i.e.,rightmost) occurrence of *b*_*j*_ in *A* that precedes position *i* of *A*, and *l*=*lastB*[*j*][*a*_*i*_] is the last occurrence of *a*_*i*_ in *B* that precedes position *j* of *B*. When *k* or *l* do not exist, case 4 of Eq. 2 does not apply.

**Fig. 1:**
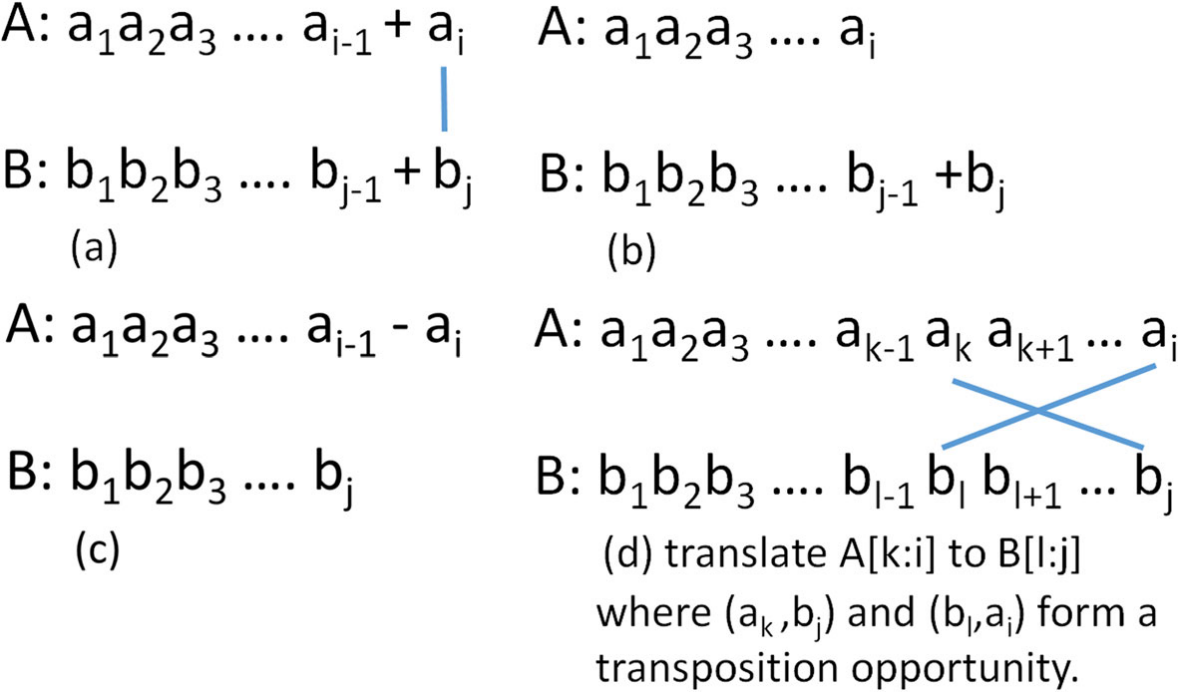
DL distance recurrence. **a** substitution. **b** insertion. **c** deletion. **d** translate A[k:i] to B[l:j] where (a _k_,b_j_) and (b _l_,a_i_) form a transposition opportunity

The four cases in Eq. 2 correspond to the four allowable edit operations: substitution, insertion, deletion and transposition. These cases are illustrated in Fig. 1, which depicts the possibilities for an optimal transformation of *A*[1:*i*] to *B*[1:*j*]. Figure 1a illustrates the first case, which is to optimally transform *A*[1:*i*−1] into *B*[1:*j*−1] and then substitute *b*_*j*_ for *a*_*i*_. If *a*_*i*_=*b*_*j*_, the substitution cost is 0, otherwise it is 1. Figure 1b shows the second case. Here, *A*[1:*i*] is optimally transformed into *B*[1:*j*−1] and then *b*_*j*_ is inserted at the end. In the third case (Fig. 1c) *A*[1:*i*−1] is optimally transformed into *B*[1:*j*] and then *a*_*i*_ is deleted. For the fourth and final case (Fig. 1d), assume that *k* and *l* exist. In this case, we are going to transpose *a*_*k*_ and *a*_*i*_. We first optimally transform *A*[1:*k*−1] into *B*[1:*l*−1]. Since only adjacent characters may be transposed, the transposition of *a*_*k*_ and *a*_*i*_ must be preceded by a deletion of *a*_*k*+1_ through *a*_*i*−1_, which results in *a*_*k*_ and *a*_*i*_ becoming adjacent. Following the transposition, we insert *b*_*l*+1_ through *b*_*j*−1_ between the transposed *a*_*k*_ and *a*_*i*_, thereby transforming *A*[1:*i*] into *B*[1:*j*]. The cost of optimally transforming *A*[1:*k*−1] into *B*[1:*l*−1] is *H*_*k*−1,*l*−1_. The ensuing deletions have a cost of *i*−*k*−1 as this is the number of deletions performed, the transposition *a*_*k*_ and *a*_*i*_ costs 1, and the final inserts cost *l*−*k*−1. So, the overall cost of case 4 is *H*_*k*−1,*l*−1_ + (*i*−*k*−1)+1 + (*j*−*l*−1).

The algorithm of Lowrance and Wagner [17] computes *H*_*m,n*_ using a *m*×*n* array for *H* and one-dimensional arrays of size *s* for *lastA* and *lastB*, where *s* is the size of the alphabet from which the strings *A* and *B* are drawn. It computes the *H*_*i,j*_’s by rows beginning with row 1 and within a row, the elements are computed left-to-right by columns. While algorithm *LS*_*DL* of [18] also computes *H* by rows and within a row by columns left-to-right, it does this using a one-dimensional array of size *n* for the current row being computed, a one-dimensional array of size *s* for *lastA*, and an *s*×*n* array *T* with the property that if *w* is the last row of *H* computed so far such that *A*[*w*]=*c*, then *T*[*c*][∗]=*H*[*w*−1][∗]. As noted in [18] when *m*<*n*, we may swap the strings *A* and *B* resulting in a space requirement of *O*(*s* min{*m,n*}+*n*). The time complexity of *LS*_*DL* is *O*(*mn*). A cache-efficient version, *Strip*_*DL*, of *LS*_*DL* that computes *H* by strips whose width is no larger than the cache size is also developed in [18]. This cache efficient algorithm has the same asymptotic time and space complexities as does *LS*_*DL*. But, as demonstrated in [18], *Strip*_*DL* is much faster than *LS*_*DL*.

The linear space algorithms we develop in this paper use a refined dynamic programming recurrence for *H*. We make the observation that when either *a*_*i*_=*b*_*j*_ or min{*i*−*k,j*−*l*}≥2 in the fourth case of Eq. 2, then it is sufficient to consider only the first three cases. To see this, note that when *a*_*i*_=*b*_*j*_ the transposition of *a*_*k*_ and *a*_*i*_ done following the deletion of *a*_*k*+1_ through *a*_*i*−1_ in case 4 (Fig. 1d) is unnecessary as *a*_*k*_ = *b*_*j*_ = *a*_*i*_. So, one of the first three cases has to result in a smaller value than case 4. Next, consider the case when *a*_*i*_≠*b*_*j*_ and min{*i*−*k,j*−*l*}≥2. Suppose that 2≤*i*−*k*≤*j*−*l*. The cost of transforming *A*[1:*i*] to *B*[1:*j*] by using an optimal transformation of *A*[1:*k*−1] to *B*[1:*l*−1] and then doing *j*−*l*+1 substitutions and inserts is *H*_*k*−1,*l*−1_+*j*−*l*+1. Doing the transformation as is case 4 has a cost *H*_*k*−1,*l*−1_+(*i*−*k*−1)+1+(*j*−*l*−1)≥*H*_*k*−1,*l*−1_+*j*−*l*+1. So, doing the transposition (case 4) isn’t any better than using only substitutions and inserts. Hence, case 4 need not be considered. The case when 2≤*j*−*l*≤*i*−*k* is symmetric.

The preceding observation establishes the correctness of the following refined recurrence for *H*.

3 $$ H_{i,0} = i,\ H_{0,j} = j, \ 0 \le i \le m, \ 0 \le j \le n  $$

When *i*>0 and *j*>0,

4 $$ {\begin{aligned} H_{i,j} = \min\left\{ \begin{array} {lcr} H_{i-1,j-1}+ c(a_{i},b_{j}) \\[-2pt] H_{i,j-1}+ 1 \\[-2pt] H_{i-1,j}+ 1 \\[-2pt] \left\{ \begin{array} {lcr} H_{k-1,j-2} + (i-k) \\[-2pt] \ \ \ \ \ \ if\ j-l=1\ and\ a_{i} \ne b_{j} \\[-2pt] H_{i-2,l-1} + (j-l) \\[-2pt] \ \ \ \ \ \ if\ i-k=1\ \ and\ a_{i} \ne b_{j} \\[-2pt] \infty \ otherwise \end{array} \right. \end{array} \right.  \end{aligned}}  $$

where *c*(*a*_*i*_,*b*_*j*_) is 1 if *a*_*i*_≠*b*_*j*_ and 0 otherwise, *k*=*lastA*[*i*][*b*_*j*_] and *l*=*lastB*[*j*][*a*_*i*_].

We observe that the above refined recurrence for *H* holds even in the weighted setting provided that 2*S*≤*I*+*D*≤2*T*, where *S*, *I*, *D*, and *T* are, respectively, the cost of a substitution, insertion, deletion, and transposition; the cost of a substitution is >0 when the characters involved are different and 0 when these are the same. This observation follows from the following.

When *a*_*i*_=*b*_*j*_,*S*=0

5 $$ \begin{aligned} &H_{k-1,l-1} + (i-k-1)D+ T + (j-l-1)I \\ &= H_{k,l} + (i-k-1)D+ T + (j-l-1)I \\ &\ge H_{i-1,l} + T + (j-l-1)I \\ &\ge H_{i-1,j-1} \\ &= H_{i,j} \end{aligned}  $$

When 2≤*i*−*k*≤*j*−*l*

6 $$ \begin{aligned} &H_{k-1,l-1} + (i-k-1)D+ T + (j-l-1)I \\ &\ge H_{k-1,l-1} + (i-k-1)(2S-I)+ S + (j-l-1)I \\ &= H_{k-1,l-1} + (i-k+1)S + ((j-l)-(i-k))I\\ &\ \ \ \ \ \ \ \ \ \ \ \ \ \ \ \ \ \ \ \ \ \ \ \ \ \ \ \ \ \ \ \ \ \ \ \ \ \ \ \ \ \ + (i-k-2)S \\ &\ge H_{k-1,l-1} + (i-k+1)S + ((j-l)-(i-k))I \\ &\ge H_{i,j} \\ \end{aligned}  $$

The case when 2≤*j*−*l*≤*i*−*k* is symmetric.

The algorithms developed in this paper are based on our refined recurrence for *H*.

## Methods

### DL distance algorithms

In this section, we develop two algorithms, *LS*_*DL*2 and *Strip*_*DL*2, to compute the DL distance between two strings of length *m* and *n* drawn from an alphabet of size *s*. We note that when *s*>*m*+*n*, at least *s*−*m*−*n* characters of the alphabet appear in neither *A* nor *B*. So, these non-appearing characters may be removed from the alphabet and we can work with this reduced size alphabet. Hence, throughout this paper, we assume that *s*≤*m*+*n*. Our algorithms, which take *O*(*m*+*n*) space, are based on the recurrence of Eqs. 3 and 4 and are the counterparts of algorithms *LS*_*DL* and *Strip*_*DL* of [18] that are based on the recurrence of Eqs. 1 and 2.

#### Algorithm *LS*_*DL*2

Like algorithm *LS*_*DL* of [18], *LS*_*DL*2 (Algorithm 1) computes *H* by rows from top to bottom and within a row by columns from left to right. For convenience, we augment *H* with row −1 and column −1. All values on this row and on this column are *maxVal*, where *maxVal* is a large value. Algorithm *LS*_*DL*2 uses 4 one-dimensional arrays *last*_*row*_*id*[1:*s*],*R*[−1:*n*],*R*1[−1:*n*], and *FR*[−1:*n*] and a few simple variables that have the following semantics when we are computing *H*_*ij*_. *k* and *l* are as in Eqs. 4.

1. *FR*[*q*]=*H*_*k*−1,*q*−2_ for the current *i* in case *q*≥*j* and for the next *i* in case *q*<*j*
2. *R*[*q*]=*H*_*i,q*_ if *q*<*j* and *H*_*i*−2,*q*_ if *q*≥*j*.
3. *R*1[*q*]=*H*_*i*−1,*q*_
4. *last*_*row*_*id*[*c*] = largest *k*<*i* such that *A*[*k*]=*c*
5. *last*_*col*_*id* = largest *l*<*j* such that *B*[*l*]=*A*[*i*]
6. *T* is the value to use for *H*_*i*−2,*l*−1_ should this be needed in the computation of *H*_*ij*_
7. *last*_*i*2*l*1=*H*_*i*−2,*j*−1_
8. *diag* ⋯ Case 1 of Eq. 4
9. *left* ⋯ Case 2 of Eq. 4
10. *up* ⋯ Case 3 of Eq. 4
11. *transpose* ⋯ Case 4 of Eq. 4

Lines 2 and 3 of the algorithm initialize *FR*, *R*1, and *R* so that following the *swap* of line 6, *R*1[*q*]=*H*_0,*q*_ and *R*[*q*]=*H*_−1,*q*_,−1≤*q*≤*n*. In other words, at the start of iteration *i*=1 of the **for** loop of lines 9-30, *R*1 and *R*, respectively, correspond to rows *i*−1 (i.e., row 0) and *i*−2 (i.e., row -1) of *H*. At this time, *FR*[−1:*n*]=*maxValue*, which corresponds to the initial situation that *k*=*lastA*[*i*][*b*_*j*_] is undefined. This will be updated as *lastA*[*i*][*b*_*j*_] gets defined. *last*_*row*_*id*[1:*s*] is initialized to −1 in line 4 for each character *c* to indicate the fact that at the start of the *i*=1 loop, *A*[*p*],*p*<1 isn’t a character of the alphabet. *last*_*col*_*id* is set to −1 at the start of each iteration of the loop of lines 5–32 as at the start of this loop, no character of *B* has been examined and so there is no last seen occurrence of *A*[*i*] in *B* (for this iteration of the **for** loop of lines 5–32). Also, at the start of the computation of each row of *H*, *last*_*i*2*l*1 is set to *R*[0], because, by the semantics of *last*_*i*2*l*1, when we are computing *H*_*i*,1_,*last*_*i*2*l*1=*H*_*i*−2,0_=*R*[0]. Following this, *R*[0] is set to *i* to indicate that the cheapest way to transform *A*[1:*i*] into *B*[0:0] is to do *i* deletes at a total cost of *i* (hence, when we are computing *H*_*i*,1_ in the loop of lines 9–30, *R*[*q*]=*H*_*ij*_,*q*<1), and *T* is set to *maxVal* as when we start a row computation, *l* is undefined. So, the initializations establish the variable semantics given above.


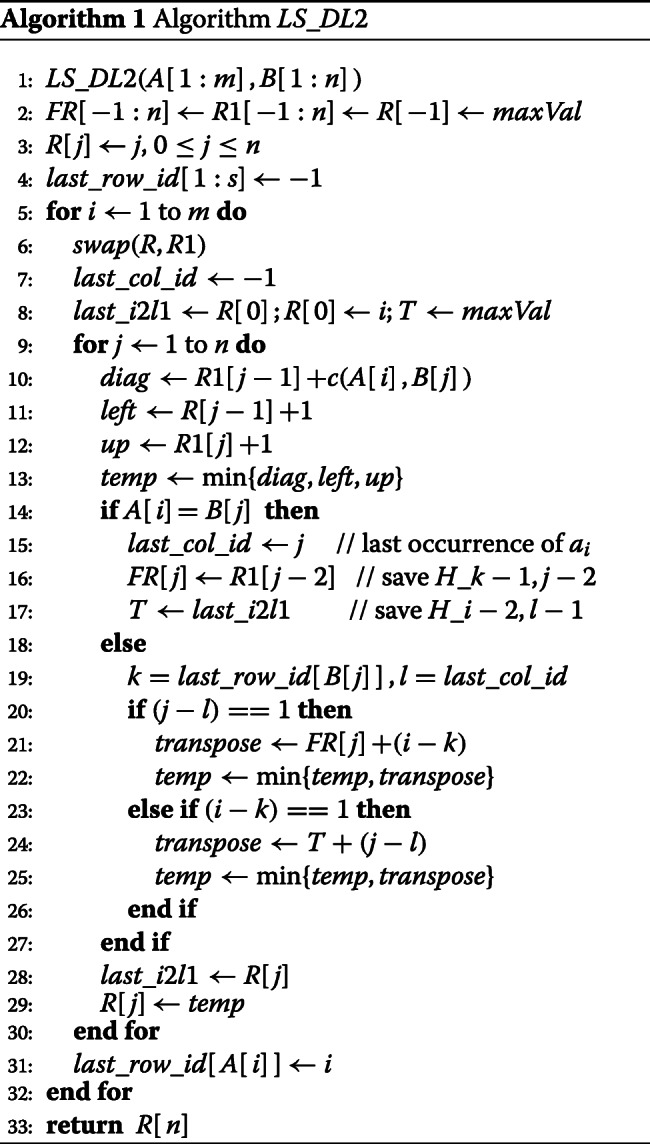


In lines 10–12 of the loop of lines 9–30, *diag*, *left*, and *up* are set to the values specified in cases 1–3 of Eq. 4. Note that from the semantics of the variables, *R*1[*j*−1]=*H*_*i*−1,*j*−1_,*R*[*j*−1]=*H*_*i,j*−1_, and *R*1[*j*]=*H*_*i*−1,*j*_ at this time. Line 13 computes the minimum of the terms in the first 3 cases of Eq. 4. If *A*[*i*]=*B*[*j*] (line 14), then *H*_*ij*_ is determined by cases 1–3 and the value of *temp* computed in line 13 is *H*_*i,j*_. At this time, we need to update *last*_*col*_*id* as the most recently seen occurrence of *A*[*i*] is now at position *j* of *B*. Since *A*[*i*]=*B*[*j*],*lastA*[*i*+1][*b*_*j*_]=*i*. So, the *H*_*k*−1,*j*−2_ to use for the next *i* in case 4 is *H*_*i*−1,*j*−2_=*R*1[*j*−2]. This value is saved in *FR*[*j*] in line 16. Since *A*[*i*]=*B*[*j*], the value to use for *H*_*i*−2,*l*−1_ in case 4 of Eq. 4 in future iterations of the **for***j* loop (until, of course, we encounter another *j* where *A*[*i*]=*B*[*j*]) becomes *H*_*i*−2,*j*−1_, which by the variable semantics is *last*_*i*2*l*1. This value is saved in *T* in line 17.

When *A*[*i*]≠*B*[*j*], lines 19–26 are executed. From the semantics of *last*_*row*_*id* and *last*_*col*_*id*, it follows that line 19 correctly sets *k* and *l*. Lines 22 and 24, respectively, compute the cost of case 4 when *j*−*l*=1 and *i*−*k*=1, respectively. Note that by the semantics of *FR* and *T*, *FR*[*j*]=*H*_*k*−1,*j*−2_ and *T*=*H*_*i*−2,*l*−1_. So, lines 22 and 25 update *temp* to be *H*_*i,j*_. When we reach line 29, regardless of whether *A*[*i*]=*B*[*j*] or *A*[*i*]≠*B*[*j*],*R*[*j*]=*H*_*i*−2,*j*_ and *temp*=*H*_*i,j*_.

In line 28, we set *last*_*i*2*l*1=*R*[*j*]=*H*_*i*−2,*j*_. While this momentarily upsets the semantics of *last*_*i*2*l*1, the semantics are restored upon the start of next iteration of the **for***j* loop as *j* increases by 1 or in line 8 if the **for***j* loop terminates and we advance to the next iteration of the **for***i* loop. Line 29 sets *R*[*j*]=*H*_*ij*_, which similarly upsets the semantics of *R* but correctly sets up the semantics for the next iteration. Finally, line 31 correctly updates *last*_*row*_*id* so as to preserve its semantics for the next iteration of the **for***i* loop.

The correctness of *LS*_*DL*2 follows from the preceding discussion. The space and time complexities are readily seen to be *O*(*m*+*n*+*s*)=*O*(*m*+*n*) and *O*(*mn*+*s*)=*O*(*mn*), respectively. When *m*<*n*, the space complexity may be reduced by a constant factor by swapping *A* and *B*. Using the LRU cache model of [18], one may show that *LS*_*DL*2 has approximately 3*mn*/*w* cache misses, where *w* is the width of a cache line. By comparison, the number of cache misses for *LS*_*DL* is *mn*(1+3/*w*).

#### Strip algorithm *Strip*_*DL*2

As in [18], we can reduce cache misses, which in turn reduces run time, by partitioning *H* into *n*/*q* strips of size *m*×*q*, where *q* is the largest strip width for which the data needed in the computation of the strip fits into the cache. *H* is computed by strips from left to right and the computation of each strip is done using *LS*_*DL*2. To enable this computation by strips, one strip needs to pass computed values to the next using three additional one-dimensional arrays *C*, *C*1, and *FC* of size *m* each. *C* records the values of *H* computed for the rightmost column in the strip; *C*1 records the values of *H* computed for the next to rightmost column in the strip; and *FC*[*i*] is the value of *T* (i.e., *H*[*i*−2][*l*−1]) at row *i*, where *l* is the last column where *B*[*l*]=*A*[*i*] in the strip. We name this new algorithm as *Strip*_*DL*2. The space complexity of *Strip*_*DL*2 is *O*(*m*+*n*) and its time complexity is *O*(*mn*). For the LRU cache model of [18] the number of cache misses is approximately $\frac {6mn}{wq}$.

### DL trace algorithms

Wagner and Fischer [19] introduced the concept of a trace to describe an edit sequence when the edit operations are limited to insert, delete, and substitute. Lowrance and Wagner [17] extended the concept of a trace to include transpositions. We reproduce here the definition and example used by us in [18]. A *trace* for the strings *A*=*a*_1_⋯*a*_*m*_ and *B*=*b*_1_⋯*b*_*n*_ is a set *T* of lines, where the endpoints *u* and *v* of a line (*u,v*) denote positions in *A* and *B*, respectively. A set of lines *T* is a trace iff:

1. For every (*u,v*)∈*T,u*≤*m* and *v*≤*n*.
2. The lines in *T* have distinct *A* positions and distinct *B* positions. That is, no two lines in *T* have the same *u* or the same *v*.

**Fig. 2:**
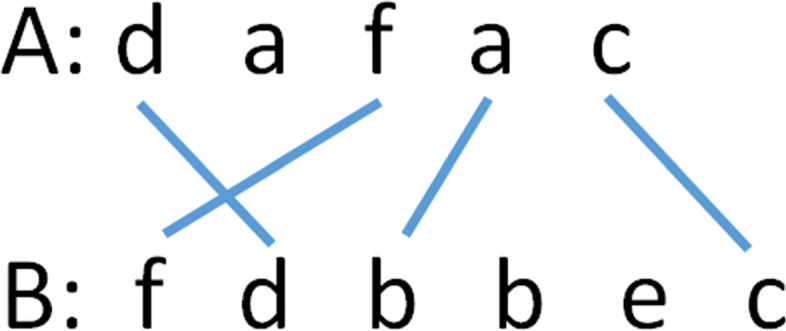
DL trace example [18]

A line (*u,v*) is *balanced* iff *a*_*u*_=*b*_*v*_ and two lines (*u*_1_,*v*_1_) and (*u*_2_,*v*_2_) cross iff (*u*_1_<*u*_2_) and (*v*_1_>*v*_2_). It is easy to see that *T*={(1,2),(3,1),(4,3),(5,6)} (see Fig. 2) is a trace for the strings *A*=*dafac* and *B*=*fdbbec*. Line (4,3) is not balanced as *a*_4_≠*b*_3_. The remaining 3 lines in the trace are balanced. The lines (1,2) and (3,1) cross.

In a trace, unbalanced lines denote a substitution operation and balanced lines denote retaining the character of *A*. If *a*_*i*_ has no line attached to it, *a*_*i*_ is to be deleted and when *b*_*j*_ has no attached line, it is to be inserted. When two balanced lines (*u*_1_,*v*_1_) and (*u*_2_,*v*_2_),*u*_1_<*u*_2_ cross, $a_{u_{1}+1} \cdots a_{u_{2}-1}$ are to be deleted from *A* making $a_{u_{1}}$ and $a_{u_{2}}$ adjacent, then $a_{u_{1}}$ and $a_{u_{2}}$ are to be transposed, and finally, $b_{v_{2}+1} \cdots b_{v_{1}-1}$ are to be inserted between the just transposed characters of *A*.

The edit sequence corresponding to the trace of Fig. 2 is delete *a*_2_, transpose *a*_1_ and *a*_3_, substitute *b* for *a*_4_, insert *b*_4_=*b* and *b*_5_=*e*, retain *a*_5_. The cost of this edit sequence is 5.

**Fig. 3:**
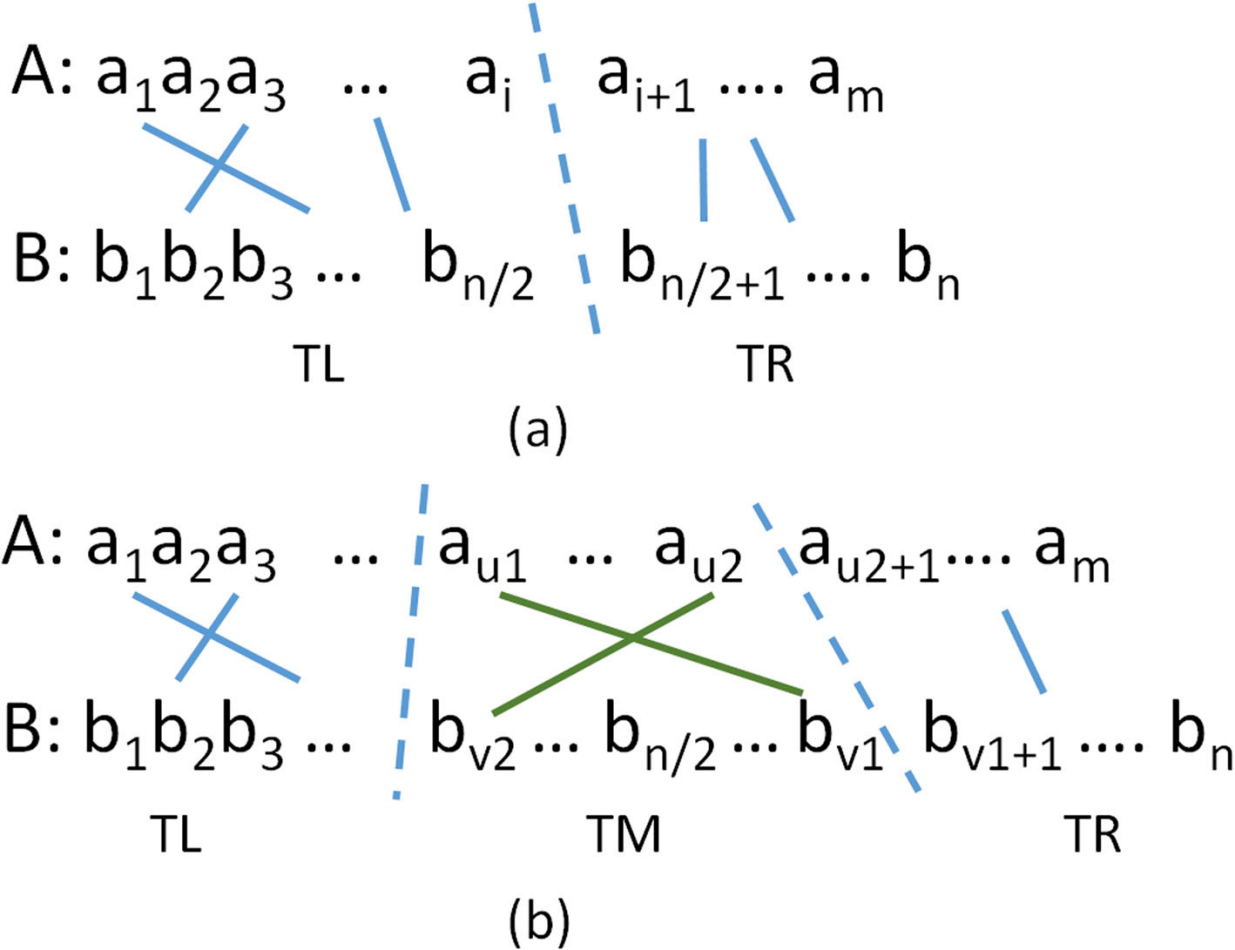
Traces with and without center crossings [18]. **a** No center crossing. **b** With center crossing

In [18], we used a divide-and-conquer strategy similar to that used by Hirschberg [20] to determine an optimal trace in *O*(*mn*) time and *O*(*s* min{*m,n*}+*n*) space. In [18], we made a distinction between traces that have a center crossing and those that do not. A trace has a *center crossing* iff it contains two lines (*u*_1_,*v*_1_) and (*u*_2_,*v*_2_) such that *v*_2_≤*n*/2 and *v*_1_>*n*/2,*u*_1_<*u*_2_, while satisfying (a) $\phantom {\dot {i}\!}a_{i} \neq a_{u_{1}} = b_{v_{1}}, u_{1} < i < u_{2}$ and (b) $\phantom {\dot {i}\!}b_{j} \neq b_{v_{2}} = a_{u_{2}}, v_{2} < j < v_{1}$. In words, *u*_1_ is the last (i.e., rightmost) occurrence of $b_{v_{1}}$ in *A* that precedes position *u*_2_ of *A* and *v*_2_ is the last occurrence of $\phantom {\dot {i}\!}a_{u_{2}}$ in *B* that precedes position *v*_1_ of *B*. (Figure 3).

In [18], we showed that the cost of an optimal trace *T* is given by Eq. 7 when *T* has no center crossing and by Eq. 8 when *T* has a center crossing. Hence, the cost of *T* is the smaller of these two costs.

7 $$  costNoCC(T) = \min_{1 \le i \le m}\{ H[i] + H'[i+1]\}  $$

where *H*[*i*] is the cost of an optimal trace for *A*[1:*i*] and *B*[1:*n*/2] and *H*^′^[*i*+1] that for an optimal trace for *A*[*i*+1:*m*] and *B*[*n*/2+1:*n*].

8 $$  {{}\begin{aligned} costCC(T) = \min\{H[u_{1}\,-\,1][v_{2}\,-\,1] + H'[u_{2}\,+\,1][v_{1}\!+1] \\[-2pt] + (u_{2}-u_{1}-1) + 1 + (v_{1}-v_{2}-1)\} \end{aligned}}  $$

where *H*[*i*][*j*] is the cost of an optimal trace for *A*[1:*i*] and *B*[1][*j*] and *H*^′^[*i*][*j*] is that for an optimal trace for *A*[*i*:*m*] and *B*[*j*][*n*]. For the min{}, we try 1≤*u*_1_<*m* and for each such *u*_1_, we set *v*_1_ to be the smallest *i*>*n*/2 for which $\phantom {\dot {i}\!}b_{i} = a_{u_{1}}$. For each *u*_1_ we examine all characters other than $a_{u_{1}}$ in the alphabet. For each such character *c*, *v*_2_ is set to the largest *j*≤*n*/2 for which *b*_*j*_=*c* and *u*_2_ is the smallest *i*>*u*_1_ for which *a*_*i*_=*c*.

Our new algorithms, *LS*_*TRACE*2 and *Strip*_*TRACE*2, are based on an adaptation of Eqs. 7 and 8 using Eq. 4.

#### Algorithm *LS*_*TRACE*2

Consider the case when the optimal trace has no center crossing. Let *R*^*f*^[] be the value of *R*[] when *LS*_*DL*2(*B*[1:*n*/2],*A*[1:*m*]) terminates and let *R*^′^^*f*^[] be the value of *R*[] when *LS*_*DL*2(*B*[*n*:*n*/2+1],*A*[*m*:1]) terminates. Let *R*1^*f*^,*R*1^′^^*f*^,*FR*^*f*^, and *FR*^′^^*f*^ be the corresponding final values for *R*1 and *RF*. From Eq. 7 and *LD*_*DL*2, we obtain

9 $$  \begin{aligned} costNoCC(T) & = \min_{1 \le i \le m}\{ H[i] + H'[i+1]\} \\ & = \min_{1 \le i \le m}\{R^{f}[i] + R'^{f}[i+1] \} \\ \end{aligned}  $$

When *T* has a center crossing {(*u*_1_,*v*_1_),(*u*_2_,*v*_2_)}, then it follows from Eq. 4 that either *u*_1_ and *u*_2_ are adjacent in *A* or *v*_1_ and *v*_2_ are adjacent in *B* (or both). When *v*_1_and *v*_2_ are adjacent in *B*, then $v_{2} = \frac {n}{2}$ and $v_{1}=\frac {n}{2}+1$. Substituting into Eq. 8, we get

10 $$ {{}\begin{aligned} costCC(T) &\,=\, \min\{H[u_{1}\,-\,1][v_{2}-1] \,+\, H'[u_{2}\,+\,1][v_{1}\,+\,1] \\ & + (u_{2}-u_{1}-1) + 1 + (v_{1}-v_{2}-1)\} \\ &= \min\{H[u_{1}\,-\,1][\frac{n}{2}\,-\,1] + H'[u_{2}\,+\,1][\frac{n}{2}+2] \\ & + (u_{2}-u_{1}) \} \\ &= \min\left\{R1^{f}[u_{1}\,-\,1] \,+\, R1'^{f}[u_{2}+1]+(u_{2}\,-\,u_{1}) \right\} \\ \end{aligned}}  $$

When *u*_1_ and *u*_2_ are adjacent in *A*, then *u*_2_−*u*_1_=1,*v*_2_ is the right most occurrence of *A*[*u*_2_] in *B* that precedes position $\frac {n}{2}+1$ (i.e., *v*_2_≤*n*/2) and *v*_1_ is the left most occurrence of *A*[*u*_1_] in *B* after position $\frac {n}{2}$ (i.e., *v*_1_≥*n*/2+1). So, we have

11 $$ {{}\begin{aligned} costCC(T) &= \min\{H[u_{1}\,-\,1][v_{2}\,-\,1] + H'[u_{2}\,+\,1][v_{1}\,+\,1] \\ & + (u_{2}-u_{1}-1) + 1 + (v_{1}-v_{2}-1)\} \\ &= \min\left\{H[u_{1}\,-\,1][v_{2}\,-\,1] \,+\, H'[u_{2}\,+\,1][v_{1}+1]\right.\\ & \left. + (v_{1}-v_{2}) \right\} \\ &\,=\, \min\left\{FR^{f}[u_{1}\,+\,1] + FR'^{f}[u_{2}\,-\,1]+ (v_{1}-v_{2}) \right\} \\ \end{aligned}}  $$

Algorithm *LS*_*TRACE*2 (Algorithm 2) provides the pseudocode for our linear space computation of an optimal trace. It assumes that *LS*_*DL*2 has been modified to return the arrays *R,R*1 and *FR*.


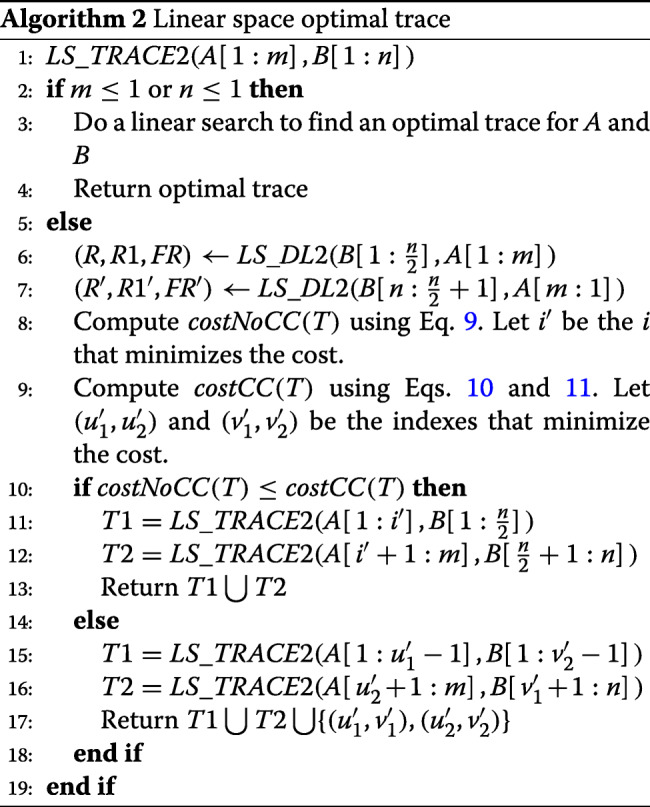


Using an analysis similar to that used by us in [18] for the analysis of *DL*_*TRACE*, we see that the time complexity of *DL*_*TRACE*2 is *O*(*mn*). The space required is the same as for *LS*_*DL*2. The number of cache misses is approximately twice that for *LS*_*DL*2 when invoked with strings of size *n* and *m*. Hence, the cache miss count for *LS*_*TRACE*2 is ≈6*mn*/*w*.

#### Strip trace algorithm *Strip*_*TRACE*2

This algorithm differs from *LS*_*TRACE*2 in that it uses a modified version of *Strip*_*DL*2 rather than a modified version of *LS*_*DL*2. The modified version of *Strip*_*DL*2 returns the arrays *C*, *C*1 and *FC* computed by *Strip*_*DL*2. The asymptotic time complexity of *Strip*_*TRACE*2 is also *O*(*mn*) and it takes the same amount of space as does *Strip*_*DL*2. The number of cache misses is approximately twice that for *Strip*_*DL*2.

## Results

We benchmarked the single-core algorithms *LS*_*DL*2,*Strip*_*DL*2,*DL*_*TRACE*2, and *Strip*_*TRACE*2 of this paper against the corresponding single-core algorithms developed by us in [18]. Using the parallelization techniques of [18], we obtained multi-core versions of our new algorithms. Their names are obtained by prefixing *PP*_ to the single-core name (e.g., *PP*_*LS*_*DL*2 is the multi-core version of *LS*_*DL*2). The new multi-core versions also were benchmarked against the corresponding multi-core algorithms of [18].

### Platforms and test data

The single-core algorithms were implemented using C and the multi-core ones using C and OpenMP. The relative performance of these algorithms was measured on the following platforms:

1. Intel Xeon CPU E5-2603 v2 Quad-Core processor 1.8GHz with 10MB cache.
2. Intel I7-x980 Six-Core processor 3.33GHz with 12MB LLC cache.
3. Intel Xeon CPU E5-2695 v2 2x12-Core processors 2.40GHz with 30MB cache.

For convenience, we will, at times, refer to these platforms as Xeon4, Xeon6, and Xeon24 (i.e., the number of cores is appended to the name Xeon).

All codes were compiled using the gcc compiler with the O2 option. On our Xeon4 platform, the benchmarking included a comparison of memory, cache misses, run time, and energy consumption. The cache miss count and the energy consumption was measured using the "perf" [21] software through the RAPL interface. For the Xeon6 and Xeon24 platforms only the run time was benchmarked.

For test data, we used randomly generated protein sequences as well as real protein sequences obtained from the Protein Data Bank [22] and DNA/RNA/protein sequences from the National Center for Biotechnology Information (NCBI) database [23]. The results for our randomly generated protein sequences were comparable to those for similarly sized sequences used from the two databases [22] and [23]. So, we present only the results for the random data sets here.

### Xeon E5-2603 (Xeon4)

#### DL distance algorithms

Table 1 gives the memory required to process random protein sequences of length 400,000 using each of the single-core DL scoring algorithms considered in this paper. LS_DL takes 4.75 times the memory taken by LS_DL2 and LS_Strip takes 4.69 times the memory taken by LS_Strip2.

**Table 1:** Memory usage for DL distance algorithms on Xeon4

Algorithm	Memory
Lowrance and Wagner	30100.88 MB
LS_ DL	41.93 MB
LS_ DL2	8.82 MB
LS_Strip	40.98 MB
LS_Strip2	8.73 MB

**Fig. 4:**
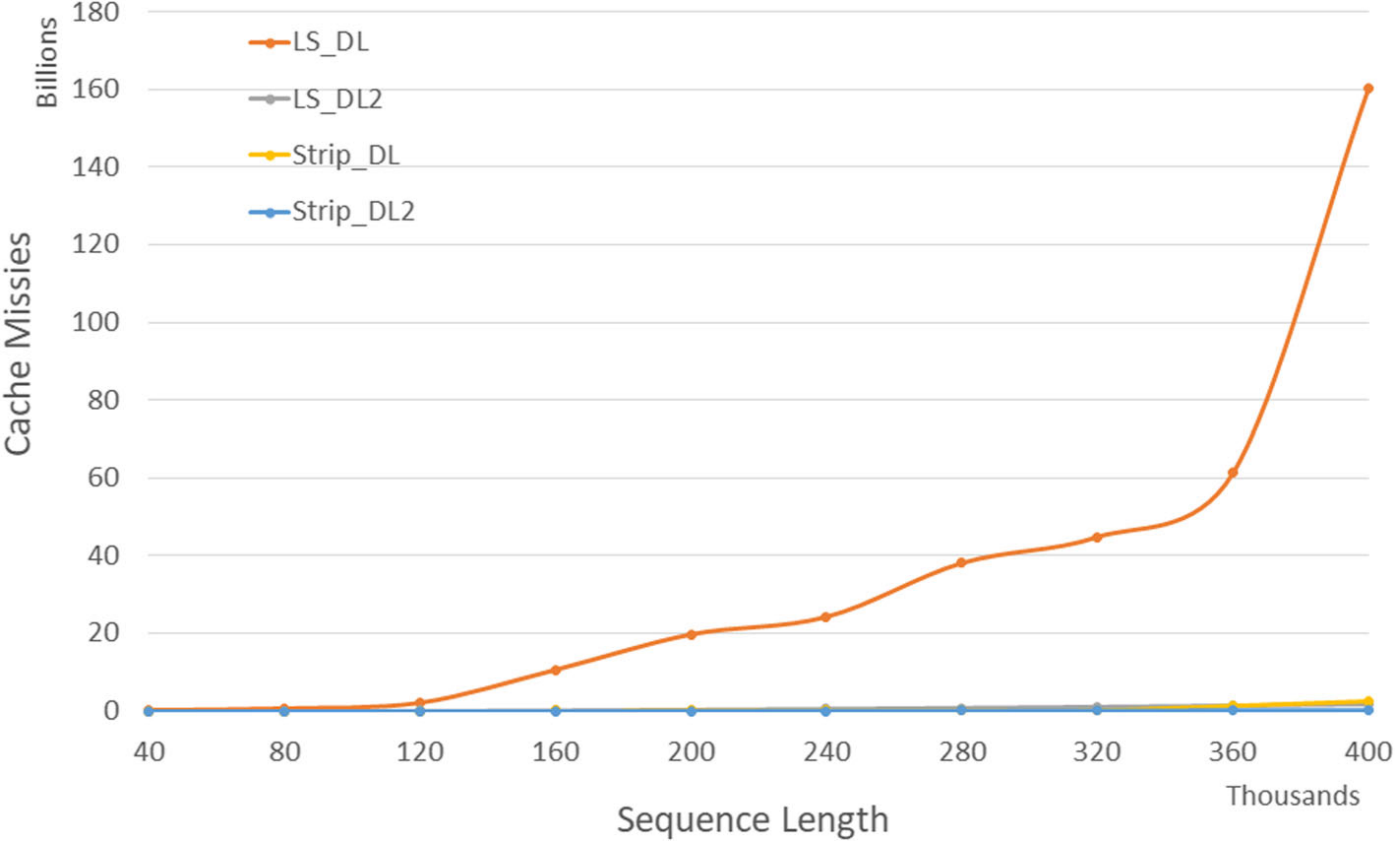
Cache misses for DL distance algorithms, in billions, on Xeon4

Figure 4 and Table 2 give the number of cache misses on our Xeon4 platform for randomly generated sequences of size between 40,000 and 400,000. The column of Table 2 labeled *LvsL*2 gives the percentage reduction in cache misses achieved by *LS*_*DL*2 relative to *LS*_*DL*, that labeled *SvsS*2 gives this percentage for *Strip*_*DL*2 relative to *Strip*_*DL*, and that labeled *L*2*vsS*2 gives this percentage for *Strip*_*DL*2 relative to *LS*_*DL*2. *Strip*_*DL*2 has the fewest cache misses. *Strip*_*DL*2 reduces cache misses by up to 91.9*%* relative to *LS*_*DL*2 and by up to 94.4*%* relative to *Strip*_*DL*.

**Table 2:** Cache misses for DL distance algorithms, in millions, on Xeon4

A	B	LS_DL	LS_DL2	Strip_DL	Strip_DL2	L vs L2	S vs S2	L2 vs S2
40000	40000	265	14	6	1	94.9%	76.6%	90.4%
80000	80000	715	53	16	5	92.6%	66.0%	89.9%
120000	120000	2,180	121	42	11	94.4%	73.1%	90.7%
160000	160000	10,652	247	63	20	97.7%	68.4%	91.9%
200000	200000	19,751	397	147	32	98.0%	78.0%	91.9%
240000	240000	24,257	570	133	49	97.7%	63.2%	91.4%
280000	280000	38,119	781	188	66	98.0%	65.0%	91.6%
320000	320000	44,815	1,021	242	86	97.7%	64.4%	91.6%
360000	360000	61,296	1,290	1,352	111	97.9%	91.8%	91.4%
400000	400000	160,118	1,587	2,407	136	99.0%	94.4%	91.5%

**Fig. 5:**
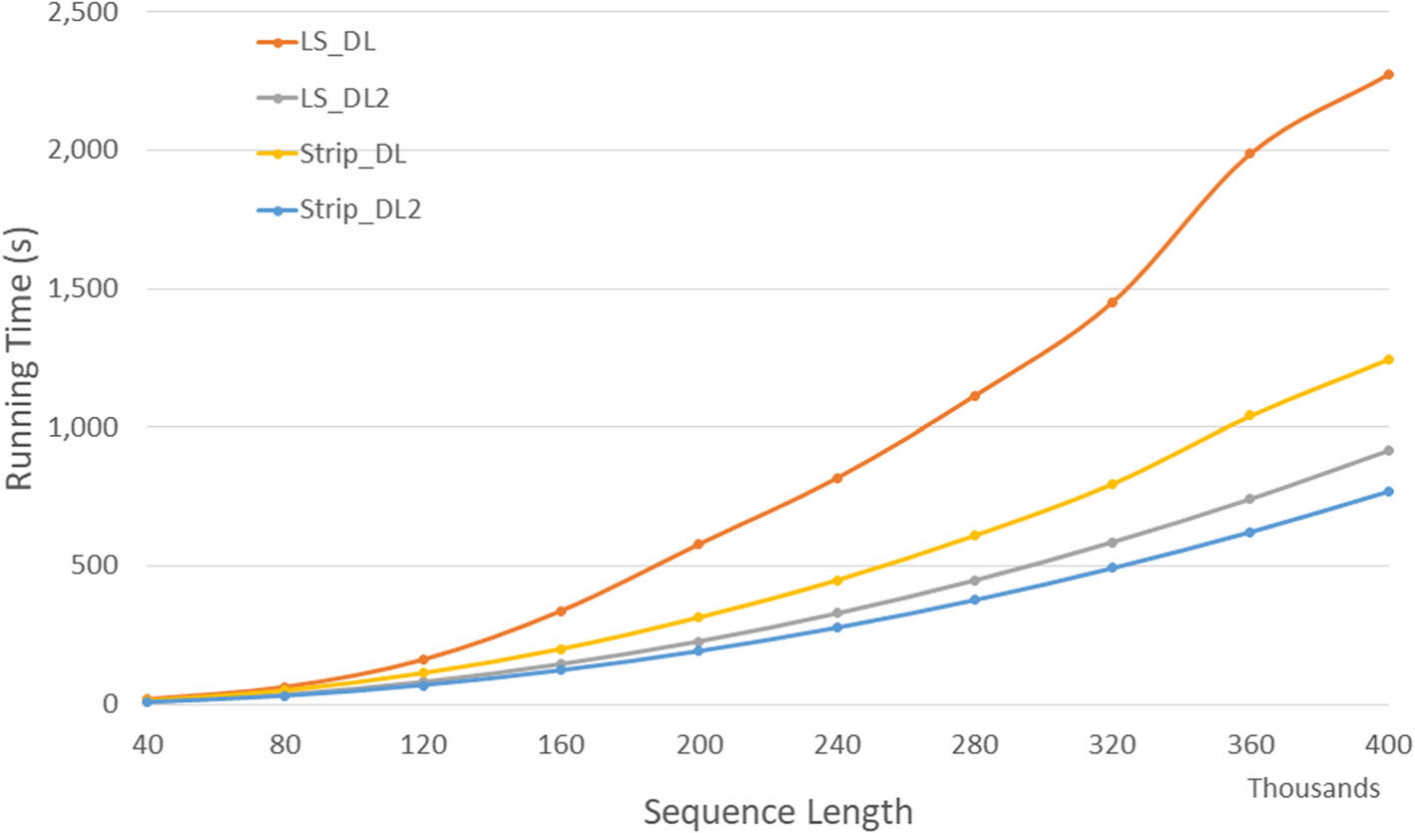
Run time for DL distance algorithms on Xeon4

Figure 5 and Table 3 give the run times on our Xeon4 platform for our random data set. In the figure, the time is in seconds while in the table, the time is given using the format *hh*:*mm*:*ss*. The table also gives the percentage reduction in run time.

**Table 3:** Run time in *hh*:*mm*:*ss* for DL distance algorithms on Xeon4

A	B	LS_DL	LS_DL2	Strip_DL	Strip_DL2	L vs L2	S vs S2	L2 vs S2
40000	40000	0:00:17	0:00:09	0:00:13	0:00:08	47.6%	38.5%	15.9%
80000	80000	0:01:02	0:00:36	0:00:50	0:00:31	41.3%	38.1%	15.4%
120000	120000	0:02:40	0:01:22	0:01:52	0:01:09	48.9%	38.3%	15.4%
160000	160000	0:05:37	0:02:26	0:03:19	0:02:03	56.6%	38.2%	15.7%
200000	200000	0:09:38	0:03:48	0:05:14	0:03:12	60.6%	38.7%	15.5%
240000	240000	0:13:37	0:05:29	0:07:28	0:04:37	59.8%	38.2%	15.7%
280000	280000	0:18:34	0:07:28	0:10:10	0:06:17	59.8%	38.2%	15.7%
320000	320000	0:24:13	0:09:45	0:13:17	0:08:13	59.7%	38.2%	15.8%
360000	360000	0:33:10	0:12:21	0:17:22	0:10:24	62.8%	40.1%	15.8%
400000	400000	0:37:55	0:15:15	0:20:46	0:12:50	59.8%	38.2%	15.8%

As can be seen, on our Xeon4 platform, *Strip*_*DL*2 is the fastest followed by *LS*_*DL*2,*Strip*_*DL*, and *LS*_*DL*. *Strip*_*DL*2 reduces run time by up to 15.9*%* relative to *LS*_*DL*2 and by up to 40.1*%* relative to *Strip*_*DL*.

**Fig. 6:**
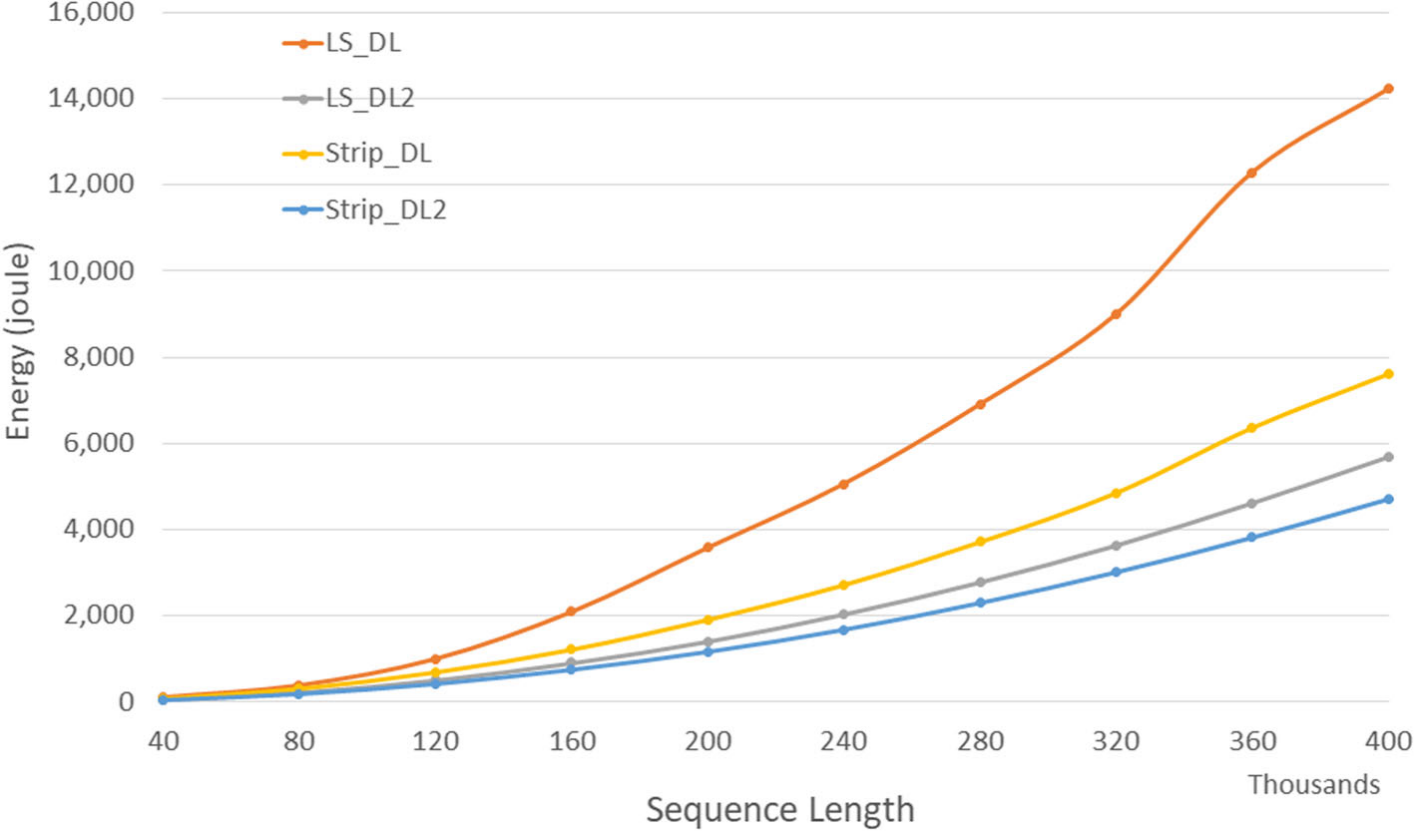
CPU/cache energy for DL distance algorithms on Xeon4

Figure 6 and Table 4 give the CPU and cache energy consumed, in joules, on our Xeon4 platform. On our data sets, *Strip*_*DL*2 required up to 17.6*%* less CPU and cache energy than *LS*_*DL*2 and up to 40.0*%* less than *Strip*_*DL*. It is interesting to note that the energy reduction is comparable to the reduction in run time suggesting a close relationship between run time and energy consumption for this application.

**Table 4:** CPU/cache energy in joules for DL distance algorithms on Xeon4

A	B	LS_DL	LS_DL2	Strip_DL	Strip_DL2	L vs L2	S vs S2	L2 vs S2
40000	40000	107	56	77	47	47.3%	39.0%	17.1%
80000	80000	384	225	305	187	41.5%	38.7%	16.7%
120000	120000	997	509	687	422	49.0%	38.5%	17.0%
160000	160000	2088	907	1212	752	56.6%	38.0%	17.1%
200000	200000	3577	1406	1906	1167	60.7%	38.8%	17.0%
240000	240000	5058	2040	2714	1680	59.7%	38.1%	17.6%
280000	280000	6906	2781	3711	2302	59.7%	38.0%	17.2%
320000	320000	9000	3638	4852	3016	59.6%	37.9%	17.1%
360000	360000	12287	4619	6366	3822	62.4%	40.0%	17.3%
400000	400000	14218	5690	7615	4712	60.0%	38.1%	17.2%

#### DL trace algorithms

**Fig. 7:**
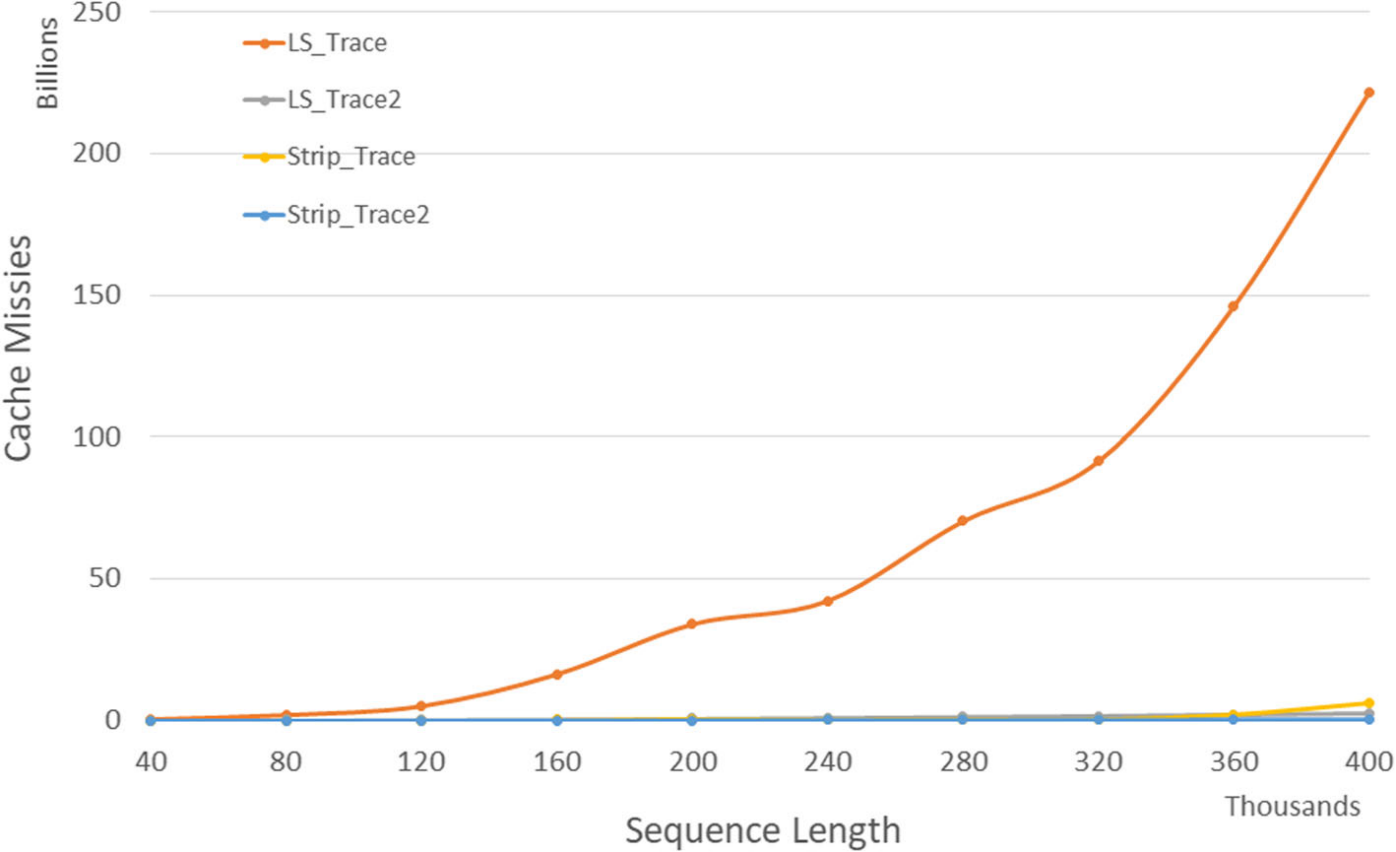
Cache misses for DL trace algorithms on Xeon4

Figure 7 and Table 5 give the number of cache misses for the trace algorithms on our Xeon4 platform for randomly generated sequences of size between 40,000 and 400,000. The column of Table 5 labeled *LvsL*2 gives the percentage reduction in cache misses achieved by *LS*_*Trace*2 relative to *LS*_*Trace*, that labeled *SvsS*2 gives this percentage *Strip*_*Trace*2 relative to *Strip*_*Trace*, and that labeled *L*2*vsS*2 gives this percentage *Strip*_*Trace*2 relative to *LS*_*Trace*2. *Strip*_*Trace*2 has the fewest cache misses. *Strip*_*Trace*2 reduces cache misses by up to 89.1*%* relative to *LS*_*Trace*2 and by up to 95.4*%* relative to *Strip*_*Trace*.

**Table 5:** Cache misses in millions for DL trace algorithms on Xeon4

A	B	LS_Trace	LS_Trace2	Strip_Trace	Strip_Trace2	L vs L2	S vs S2	L2 vs S2
40000	40000	423	20	24	3	95.2%	88.1%	86.2%
80000	80000	1,970	89	29	12	95.5%	58.5%	86.6%
120000	120000	5,100	212	66	25	95.8%	61.8%	88.1%
160000	160000	16,350	403	115	44	97.5%	61.9%	89.1%
200000	200000	33,998	622	513	72	98.2%	85.9%	88.3%
240000	240000	42,252	897	268	101	97.9%	62.4%	88.8%
280000	280000	70,370	1,244	358	139	98.2%	61.3%	88.9%
320000	320000	91,501	1,576	453	181	98.3%	60.1%	88.5%
360000	360000	146,103	2,001	2,120	230	98.6%	89.1%	88.5%
400000	400000	221,690	2,435	6,032	276	98.9%	95.4%	88.6%

**Fig. 8:**
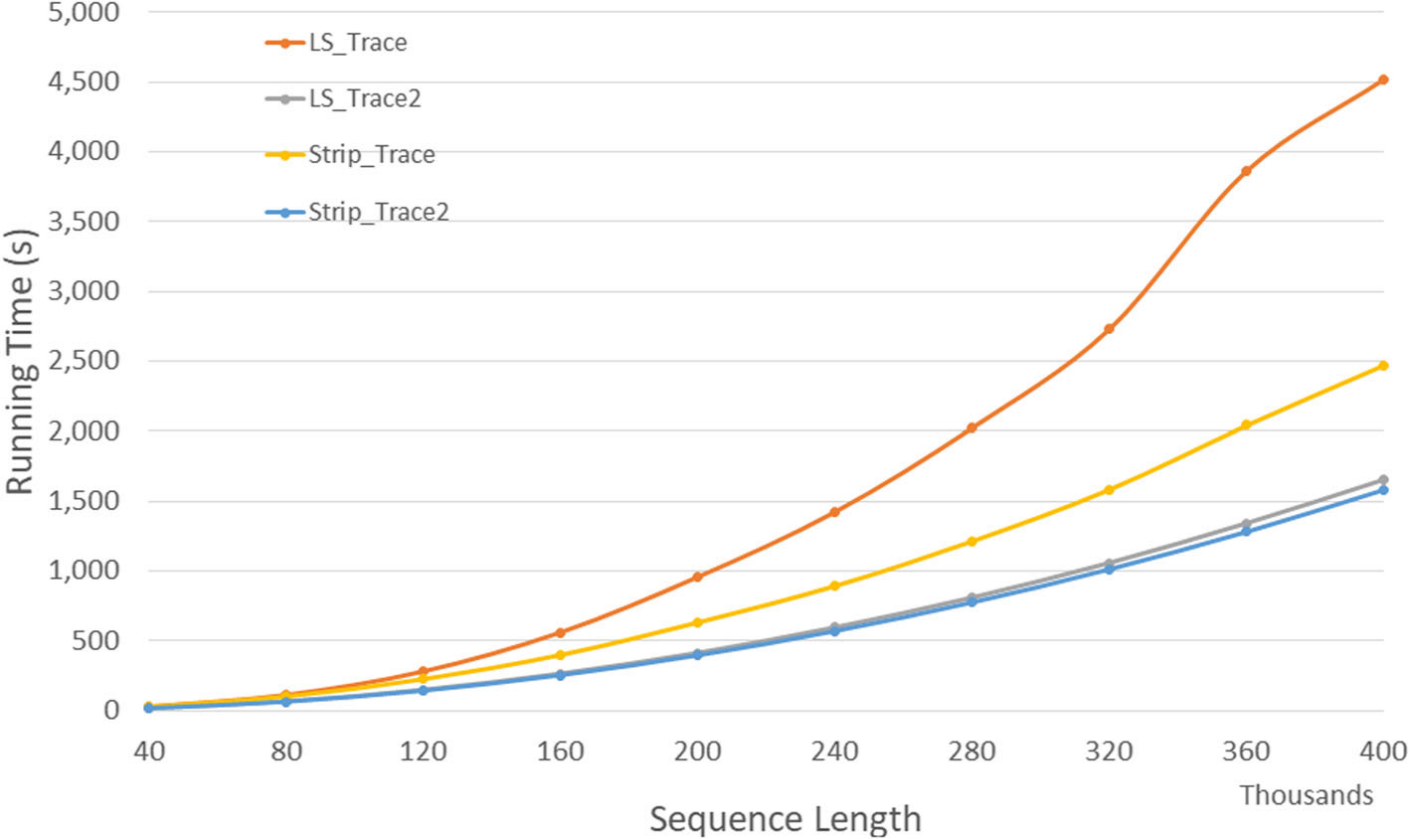
Run time for DL trace algorithms on Xeon4

Figure 8 and Table 6 give the run times on our Xeon4 platform for our random data set. The table also gives the percentage reduction in run time. *Strip*_*Trace*2 is the fastest followed by *LS*_*Trace*2,*Strip*_*Trace*, and *LS*_*Trace* (in this order). *Strip*_*Trace*2 reduces run time by up to 4.3*%* relative to *LS*_*Trace*2 and by up to 37.8*%* relative to *Strip*_*Trace*.

**Table 6:** Run time in *hh*:*mm*:*ss* for DL trace algorithms on Xeon4

A	B	LS_Trace	LS_Trace2	Strip_Trace	Strip_Trace2	L vs L2	S vs S2	L2 vs S2
40000	40000	0:00:30	0:00:17	0:00:26	0:00:16	44.0%	37.8%	3.6%
80000	80000	0:01:54	0:01:06	0:01:40	0:01:04	42.1%	36.5%	3.8%
120000	120000	0:04:42	0:02:29	0:03:44	0:02:23	47.3%	36.2%	4.0%
160000	160000	0:09:21	0:04:25	0:06:37	0:04:14	52.8%	36.1%	4.2%
200000	200000	0:15:58	0:06:53	0:10:30	0:06:36	56.8%	37.1%	4.1%
240000	240000	0:23:42	0:09:56	0:14:52	0:09:31	58.1%	36.0%	4.2%
280000	280000	0:33:41	0:13:29	0:20:13	0:12:57	60.0%	36.0%	4.1%
320000	320000	0:45:26	0:17:37	0:26:24	0:16:54	61.2%	36.0%	4.1%
360000	360000	1:04:18	0:22:21	0:34:01	0:21:23	65.2%	37.1%	4.3%
400000	400000	1:15:14	0:27:33	0:41:11	0:26:24	63.4%	35.9%	4.2%

**Fig. 9:**
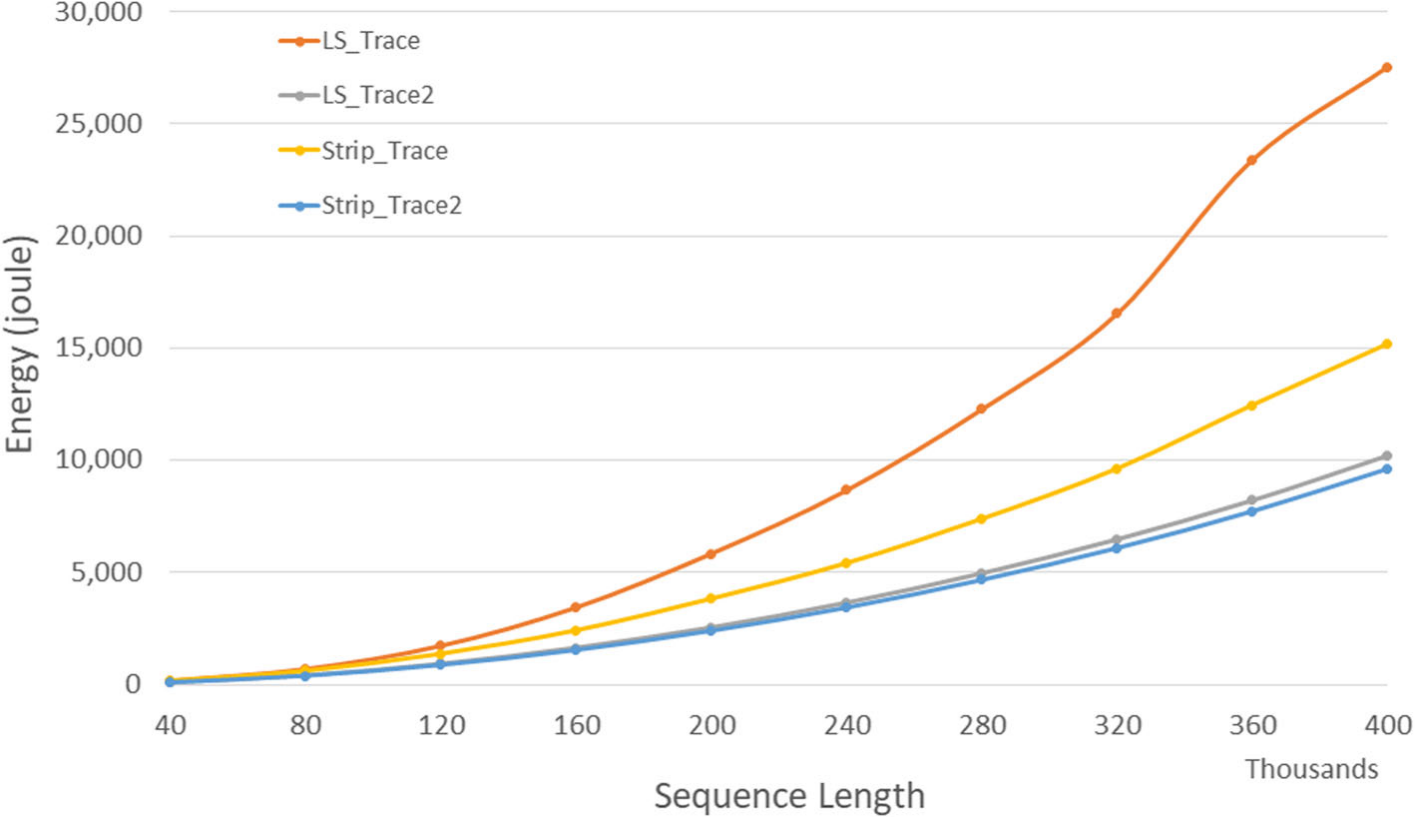
CPU/cache energy for DL trace algorithms on Xeon4

Figure 9 and Table 7 give the CPU and cache energy consumed, in joules, *Strip*_*Trace*2 required up to 6.3*%* less CPU and cache energy than *LS*_*Trace*2 and up to 37.9*%* less than *Strip*_*Trace*.

**Table 7:** CPU/cache energy in joules for DL trace algorithms on Xeon4

A	B	LS_Trace	LS_Trace2	Strip_Trace	Strip_Trace2	L vs L2	S vs S2	L2 vs S2
40000	40000	181.4	103.1	157.0	97.8	43.2%	37.7%	5.2%
80000	80000	703.1	413.8	610.7	389.3	41.1%	36.3%	5.9%
120000	120000	1,736.9	929.9	1,365.3	873.4	46.5%	36.0%	6.1%
160000	160000	3,443.8	1,644.4	2,407.9	1,540.3	52.3%	36.0%	6.3%
200000	200000	5,843.6	2,556.4	3,818.7	2,398.1	56.3%	37.2%	6.2%
240000	240000	8,665.3	3,659.6	5,403.6	3,430.8	57.8%	36.5%	6.3%
280000	280000	12,275.0	4,970.5	7,372.3	4,665.3	59.5%	36.7%	6.1%
320000	320000	16,536.9	6,486.5	9,609.6	6,084.6	60.8%	36.7%	6.2%
360000	360000	23,396.4	8,224.1	12,439.7	7,725.9	64.8%	37.9%	6.1%
400000	400000	27,551.9	10,229.2	15,167.8	9,616.6	62.9%	36.6%	6.0%

#### Parallel algorithms

**Fig. 10:**
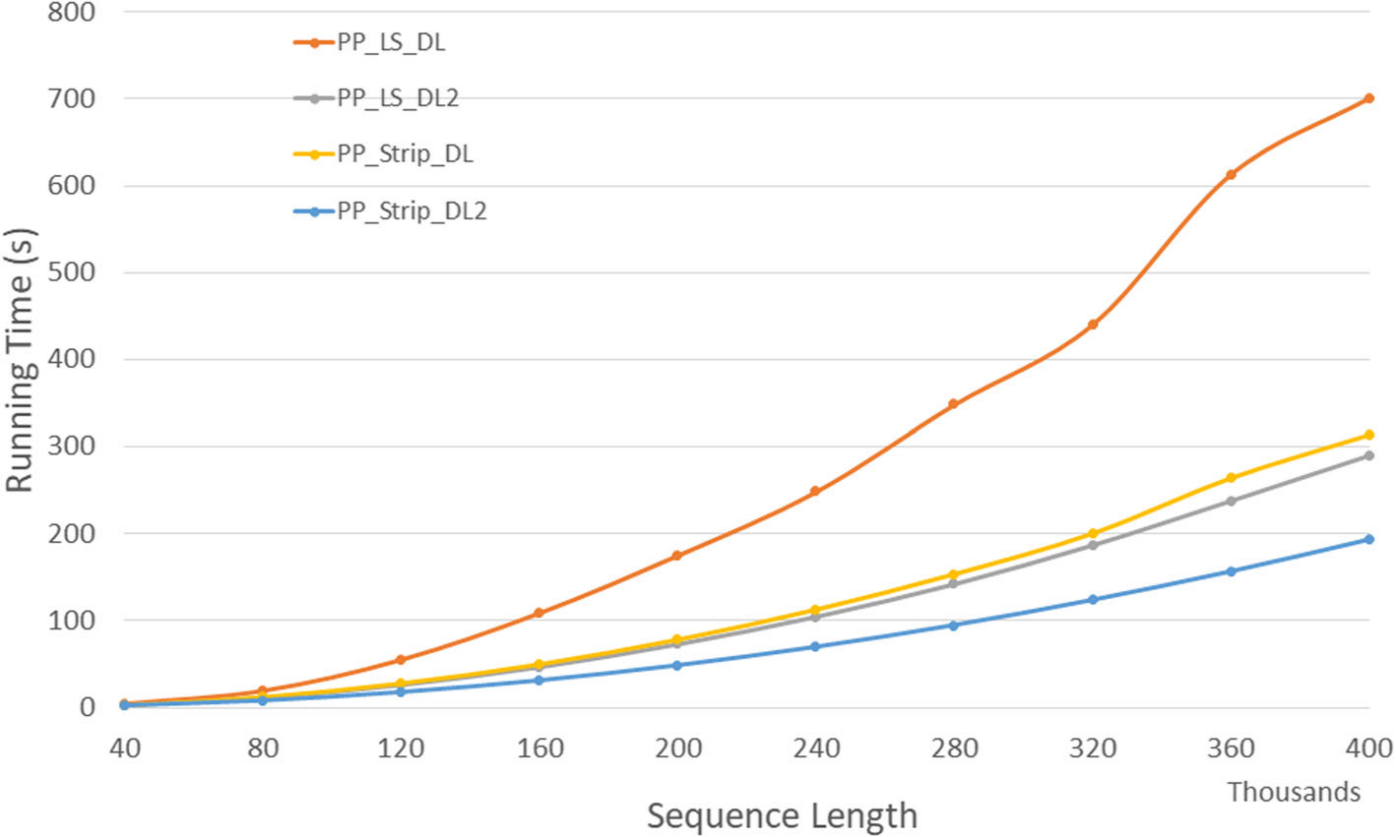
Run time of parallel DL distance algorithms, in seconds, on Xeon4

Figure 10 and Table 8 give the run times for our parallel DL distance algorithms on our Xeon4 platform. *PP*_*LS*_*DL*2 is up to 61.2*%* faster than *PP*_*LS*_*DL* and *PP*_*Strip*_*DL*2 is up to 40.6*%* faster than *PP*_*Strip*_*DL*. Also, *PP*_*LS*_*DL*2 and *PP*_*Strip*_*DL*2 achieve a speedup of up to 3.15 and 3.98 compared to the corresponding single-core algorithms on a four-core machine, respectively.

**Table 8:** Run time of parallel DL distance algorithms, in *hh*:*mm*:*ss*, on Xeon4

A	B	PP_LS_DL	PP_LS_DL2	PP_Strip_DL	PP_Strip_DL2	L vs L2	S vs S2	L2 vs S2
40000	40000	0:00:05	0:00:03	0:00:03	0:00:02	42.4%	38.1%	33.8%
80000	80000	0:00:20	0:00:12	0:00:13	0:00:08	41.8%	38.1%	33.6%
120000	120000	0:00:56	0:00:26	0:00:28	0:00:17	52.8%	38.5%	33.8%
160000	160000	0:01:49	0:00:47	0:00:50	0:00:31	57.2%	38.3%	33.9%
200000	200000	0:02:55	0:01:13	0:01:19	0:00:48	58.3%	38.4%	33.9%
240000	240000	0:04:09	0:01:45	0:01:53	0:01:10	57.9%	38.3%	33.5%
280000	280000	0:05:48	0:02:22	0:02:34	0:01:35	59.1%	38.3%	33.4%
320000	320000	0:07:21	0:03:07	0:03:20	0:02:04	57.5%	38.2%	33.8%
360000	360000	0:10:13	0:03:58	0:04:24	0:02:37	61.2%	40.6%	34.2%
400000	400000	0:11:41	0:04:50	0:05:13	0:03:14	58.6%	38.2%	33.3%

**Fig. 11:**
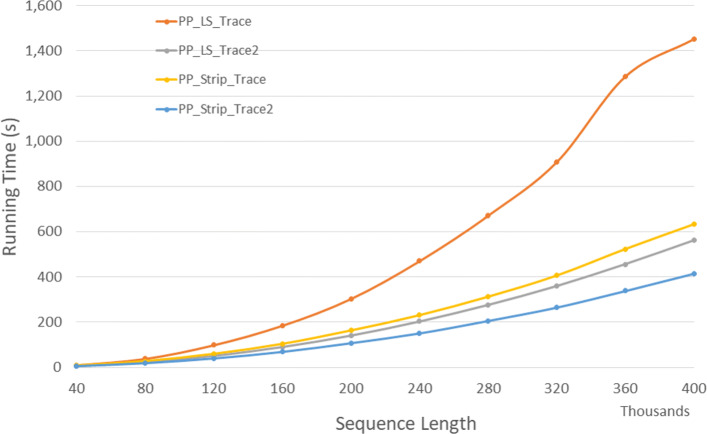
Run time of parallel DL trace algorithms, in seconds, on Xeon4

Figure 11 and Table 9 give the run times for our parallel DL trace algorithms on our Xeon4 platform. *PP*_*LS*_*Trace*2 is up to 64.5*%* faster than *PP*_*LS*_*Trace* and *PP*_*Strip*_*Trace*2 is up to 35.4*%* faster than *PP*_*Strip*_*Trace*. Also, *PP*_*LS*_*Trace*2 and *PP*_*Strip*_*Trace*2 achieves a speedup up to 2.94 and 3.83 compared to the corresponding single-core algorithms, respectively.

**Table 9:** Run time of parallel DL trace algorithms, in *hh*:*mm*:*ss*, on Xeon4

A	B	PP_LS_Trace	PP_LS_Trace2	PP_Strip_Trace	PP_Strip_Trace2	L vs L2	S vs S2	L2 vs S2
40000	40000	0:00:10	0:00:06	0:00:07	0:00:05	40.8%	27.1%	10.4%
80000	80000	0:00:38	0:00:23	0:00:27	0:00:19	39.9%	30.7%	19.2%
120000	120000	0:01:39	0:00:51	0:00:59	0:00:40	48.3%	33.0%	22.6%
160000	160000	0:03:05	0:01:31	0:01:43	0:01:09	51.0%	33.5%	24.2%
200000	200000	0:05:03	0:02:21	0:02:43	0:01:47	53.5%	34.2%	24.0%
240000	240000	0:07:50	0:03:23	0:03:50	0:02:30	56.8%	34.7%	26.0%
280000	280000	0:11:11	0:04:36	0:05:13	0:03:26	58.8%	34.2%	25.6%
320000	320000	0:15:07	0:06:00	0:06:46	0:04:25	60.3%	34.8%	26.4%
360000	360000	0:21:25	0:07:37	0:08:44	0:05:38	64.5%	35.4%	25.9%
400000	400000	0:24:10	0:09:22	0:10:34	0:06:55	61.3%	34.6%	26.1%

### I7-x980 (Xeon6)

#### DL distance algorithms

**Fig. 12:**
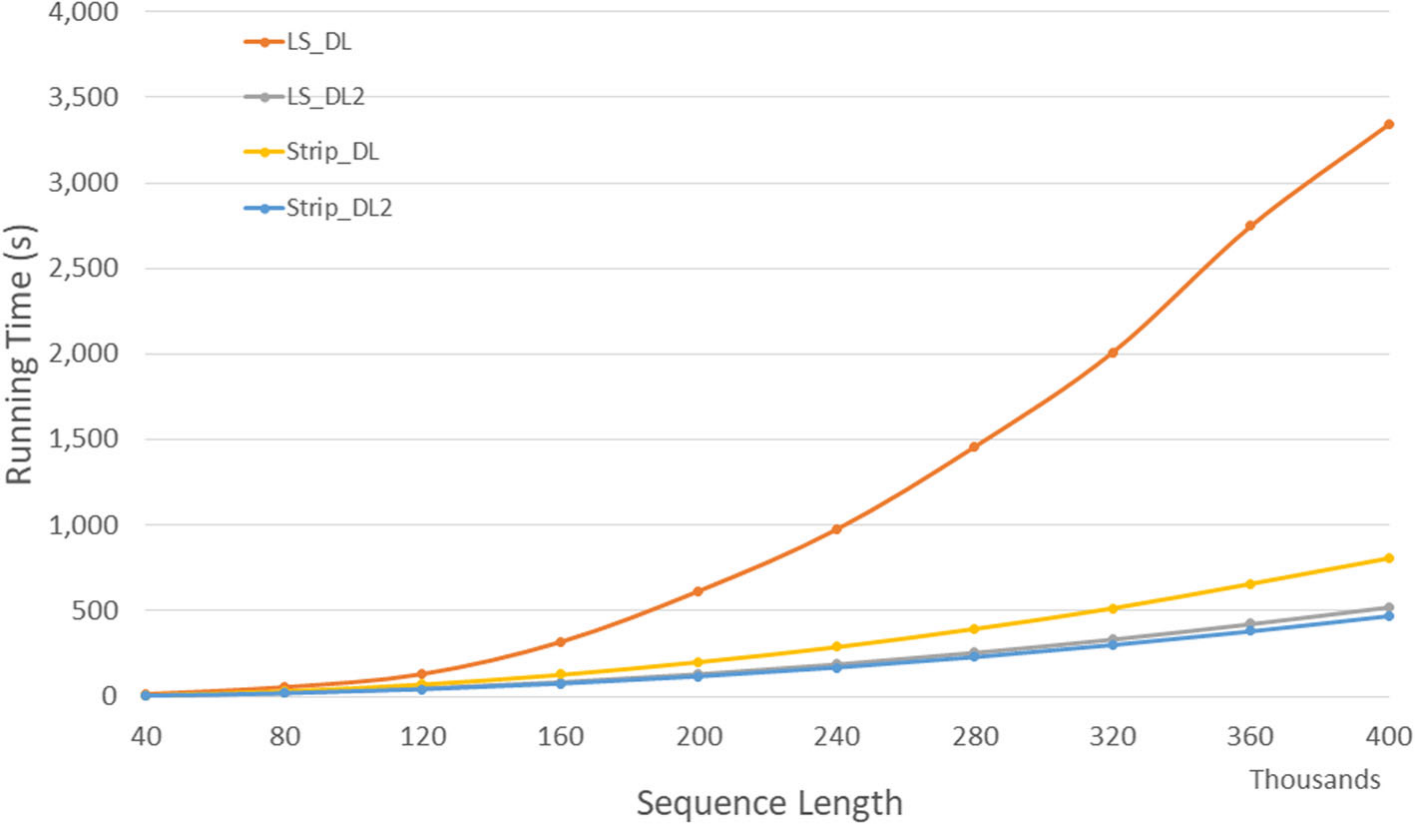
Run time for DL distance algorithms on Xeon6

Figure 12 and Table 10 give the run times of our single-core distance algorithms on our Xeon6 platform. As can be seen, *Strip*_*DL*2 is the fastest followed by *LS*_*DL*2,*Strip*_*DL*, and *LS*_*DL* (in this order). *Strip*_*DL*2 reduces run time by up to 10.2*%* relative to *LS*_*DL*2 and by up to 42.0*%* relative to *Strip*_*DL*.

**Table 10:** Run time in *hh*:*mm*:*ss* for DL distance algorithms on Xeon6

A	B	LS_DL	LS_DL2	Strip_DL	Strip_DL2	L vs L2	S vs S2	L2 vs S2
40000	40000	0:00:14	0:00:05	0:00:08	0:00:05	63.2%	41.7%	9.8%
80000	80000	0:00:55	0:00:21	0:00:32	0:00:19	61.9%	41.5%	9.9%
120000	120000	0:02:12	0:00:47	0:01:13	0:00:42	64.3%	41.5%	9.7%
160000	160000	0:05:19	0:01:24	0:02:09	0:01:15	73.7%	41.5%	9.9%
200000	200000	0:10:16	0:02:11	0:03:23	0:01:58	78.7%	41.8%	9.9%
240000	240000	0:16:17	0:03:09	0:04:50	0:02:50	80.7%	41.5%	10.0%
280000	280000	0:24:19	0:04:17	0:06:36	0:03:51	82.4%	41.7%	10.1%
320000	320000	0:33:32	0:05:35	0:08:36	0:05:02	83.3%	41.5%	10.0%
360000	360000	0:45:50	0:07:06	0:10:58	0:06:22	84.5%	42.0%	10.2%
400000	400000	0:55:44	0:08:44	0:13:27	0:07:52	84.3%	41.6%	10.1%

#### DL trace algorithms

**Fig. 13:**
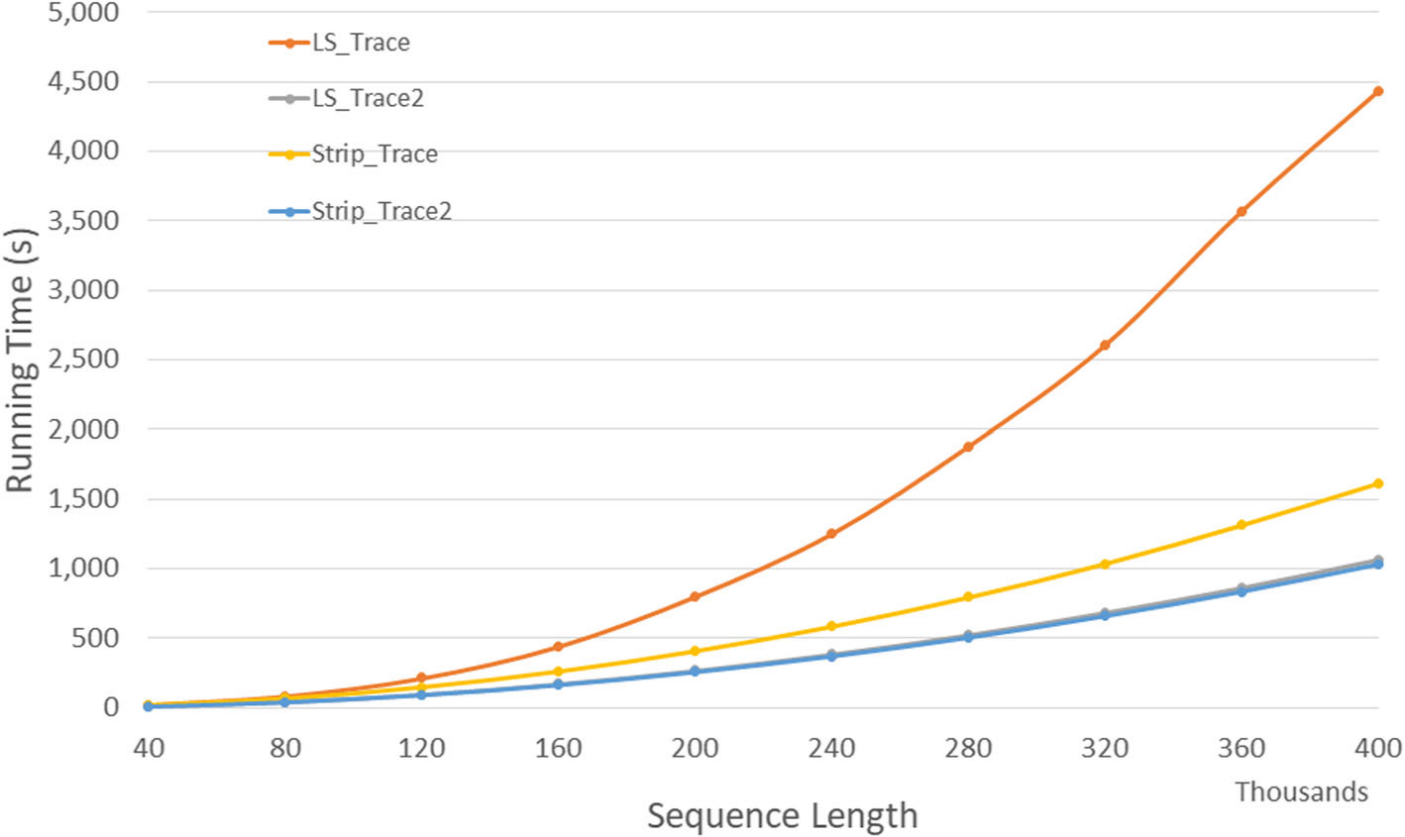
Run time for DL trace algorithms on Xeon6

Figure 13 and Table 11 give the run times of our single-core trace algorithms on our Xeon6 platform. *Strip*_*Trace*2 is the fastest followed by *LS*_*Trace*2,*Strip*_*Trace*, and *LS*_*Trace* (in this order). *Strip*_*Trace*2 reduces run time by up to 3.9*%* relative to *LS*_*Trace*2 and by up to 37.6*%* relative to *Strip*_*Trace*.

**Table 11:** Run time in *hh*:*mm*:*ss* for DL trace algorithms on Xeon6

A	B	LS_Trace	LS_Trace2	Strip_Trace	Strip_Trace2	L vs L2	S vs S2	L2 vs S2
40000	40000	0:00:22	0:00:11	0:00:17	0:00:10	51.4%	37.6%	3.2%
80000	80000	0:01:24	0:00:43	0:01:05	0:00:41	49.1%	36.9%	3.5%
120000	120000	0:03:33	0:01:36	0:02:26	0:01:33	54.9%	36.8%	3.6%
160000	160000	0:07:20	0:02:51	0:04:20	0:02:44	61.2%	36.7%	3.8%
200000	200000	0:13:19	0:04:27	0:06:46	0:04:17	66.6%	36.8%	3.9%
240000	240000	0:20:51	0:06:24	0:09:43	0:06:10	69.3%	36.6%	3.8%
280000	280000	0:31:19	0:08:43	0:13:14	0:08:23	72.1%	36.6%	3.8%
320000	320000	0:43:24	0:11:24	0:17:16	0:10:57	73.7%	36.6%	3.9%
360000	360000	0:59:27	0:14:23	0:21:55	0:13:52	75.8%	36.8%	3.7%
400000	400000	1:13:51	0:17:47	0:26:57	0:17:07	75.9%	36.5%	3.8%

#### Parallel algorithms

**Fig. 14:**
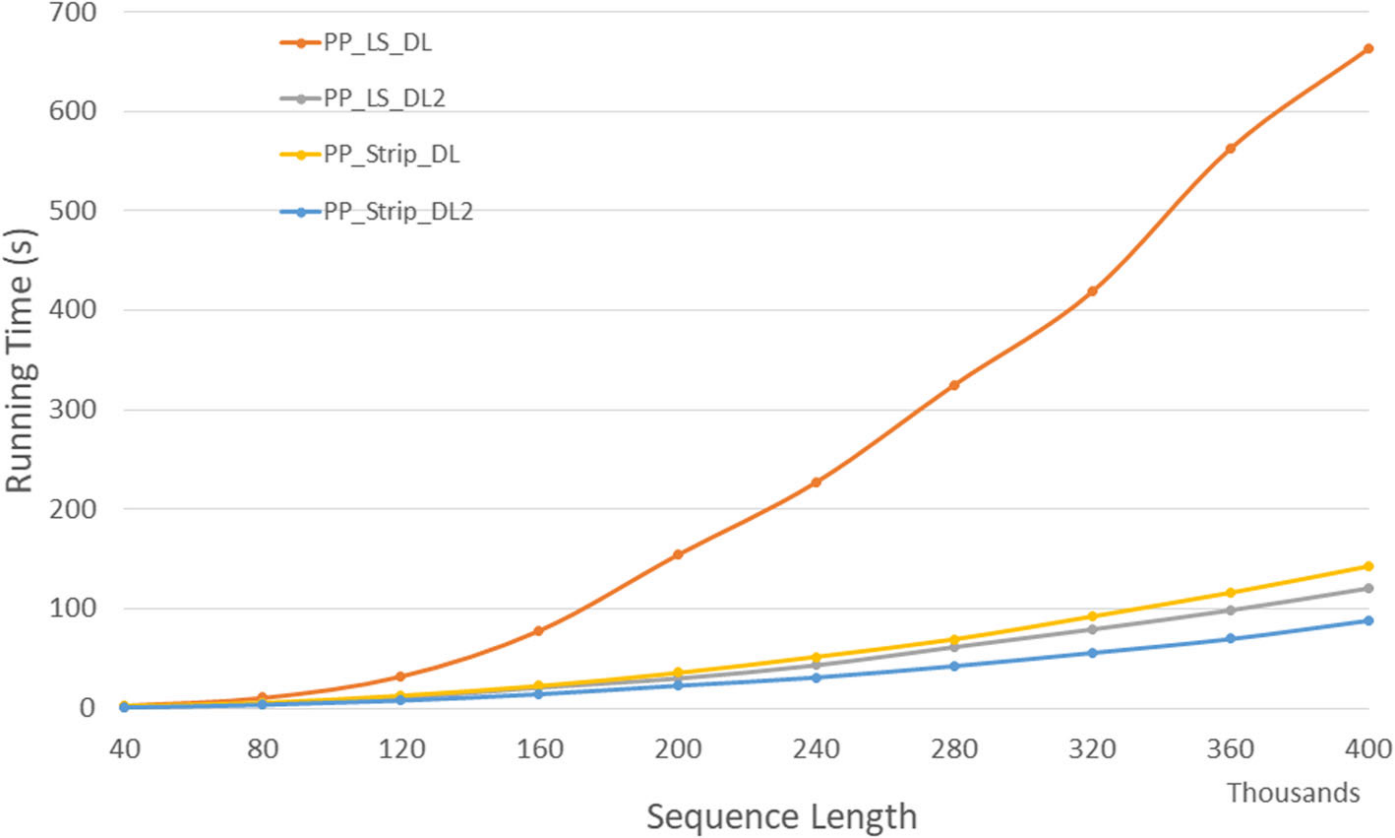
Run time of parallel DL distance algorithms, in seconds, on Xeon6

Figure 14 and Table 12 give the run times for our parallel DL distance algorithms on our Xeon6 platform. *PP*_*LS*_*DL*2 is up to 82.4*%* faster than *PP*_*LS*_*DL* and *PP*_*Strip*_*DL*2 is up to 39.8*%* faster than *PP*_*Strip*_*DL*. Also, *PP*_*LS*_*DL*2 and *PP*_*Strip*_*DL*2 achieves a speedup up to 4.32 and 5.44 compared to the corresponding single core algorithms on a six core machine, respectively.

**Table 12:** Run time of parallel DL distance algorithms, in *hh*:*mm*:*ss*, on Xeon6

A	B	PP_LS_DL	PP_LS_DL2	PP_Strip_DL	PP_Strip_DL2	L vs L2	S vs S2	L2 vs S2
40000	40000	0:00:03	0:00:01	0:00:03	0:00:01	54.6%	64.1%	24.4%
80000	80000	0:00:11	0:00:05	0:00:06	0:00:04	54.2%	37.4%	27.2%
120000	120000	0:00:32	0:00:11	0:00:13	0:00:08	66.1%	39.4%	28.1%
160000	160000	0:01:18	0:00:21	0:00:23	0:00:14	72.8%	36.4%	32.5%
200000	200000	0:02:34	0:00:30	0:00:36	0:00:23	80.3%	35.9%	24.4%
240000	240000	0:03:47	0:00:44	0:00:52	0:00:31	80.8%	39.8%	28.5%
280000	280000	0:05:25	0:01:02	0:01:09	0:00:43	80.9%	38.4%	31.0%
320000	320000	0:06:59	0:01:20	0:01:32	0:00:56	80.9%	39.2%	29.6%
360000	360000	0:09:23	0:01:39	0:01:56	0:01:10	82.4%	39.6%	29.2%
400000	400000	0:11:03	0:02:01	0:02:23	0:01:29	81.7%	37.8%	26.7%

**Fig. 15:**
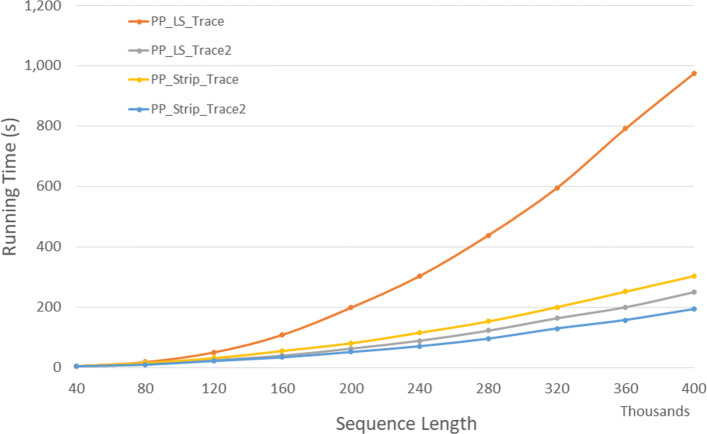
Run time of parallel DL trace algorithms, in seconds, on Xeon6

Figure 15 and Table 13 give the run times for our parallel DL trace algorithms on our Xeon6 platform. *PP*_*LS*_*Trace*2 is up to 74.7*%* faster than PP_LS_Trace and *PP*_*Strip*_*Trace*2 is up to 41.8*%* faster than *PP*_*Strip*_*Trace*. Also, *PP*_*LS*_*Trace*2 and *PP*_*Strip*_*Trace*2 achieves a speedup up to 4.32 and 5.30 compared to the corresponding single-core algorithms, respectively.

**Table 13:** Run time of parallel DL trace algorithms, in *hh*:*mm*:*ss*, on Xeon6

A	B	PP_LS_Trace	PP_LS_Trace2	PP_Strip_Trace	PP_Strip_Trace2	L vs L2	S vs S2	L2 vs S2
40000	40000	0:00:05	0:00:05	0:00:05	0:00:04	10.3%	20.6%	14.3%
80000	80000	0:00:19	0:00:10	0:00:16	0:00:09	44.8%	41.8%	11.0%
120000	120000	0:00:51	0:00:25	0:00:31	0:00:21	51.0%	31.8%	13.8%
160000	160000	0:01:49	0:00:40	0:00:56	0:00:33	63.3%	39.9%	16.3%
200000	200000	0:03:19	0:01:03	0:01:21	0:00:51	68.4%	36.7%	18.7%
240000	240000	0:05:04	0:01:29	0:01:56	0:01:10	70.7%	39.2%	21.0%
280000	280000	0:07:19	0:02:02	0:02:33	0:01:36	72.1%	37.6%	21.9%
320000	320000	0:09:55	0:02:43	0:03:21	0:02:09	72.5%	35.5%	20.8%
360000	360000	0:13:11	0:03:20	0:04:12	0:02:38	74.7%	37.4%	21.3%
400000	400000	0:16:14	0:04:10	0:05:03	0:03:14	74.3%	36.1%	22.5%

### Xeon E5-2695 (Xeon24)

#### DL distance algorithms

**Fig. 16:**
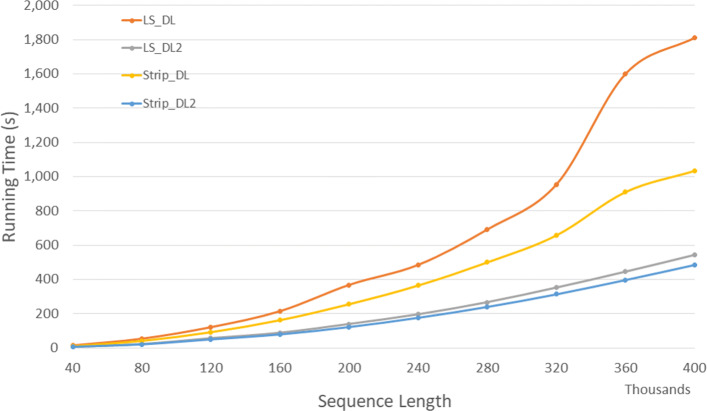
Run time for DL distance algorithms on Xeon24

Figure 16 and Table 14 give the run times of our single-core distance algorithms on our Xeon24 platform. *Strip*_*DL*2 is the fastest followed by *LS*_*DL*2,*Strip*_*DL*, and *LS*_*DL* (in this order). *Strip*_*DL*2 reduces run time by up to 13.8*%* relative to *LS*_*DL*2 and by up to 56.4*%* relative to *Strip*_*DL*.

**Table 14:** Run time in *hh*:*mm*:*ss* for DL distance algorithms on Xeon24

A	B	LS_DL	LS_DL2	Strip_DL	Strip_DL2	L vs L2	S vs S2	L2 vs S2
40000	40000	0:00:14	0:00:06	0:00:10	0:00:05	58.0%	50.6%	13.8%
80000	80000	0:00:53	0:00:22	0:00:41	0:00:20	58.1%	51.6%	11.9%
120000	120000	0:02:01	0:00:57	0:01:31	0:00:49	53.1%	46.5%	13.5%
160000	160000	0:03:34	0:01:28	0:02:42	0:01:18	58.8%	51.9%	11.6%
200000	200000	0:06:08	0:02:19	0:04:15	0:02:01	62.1%	52.4%	12.9%
240000	240000	0:08:05	0:03:16	0:06:05	0:02:56	59.6%	51.8%	10.4%
280000	280000	0:11:32	0:04:27	0:08:20	0:03:59	61.5%	52.1%	10.2%
320000	320000	0:15:55	0:05:52	0:10:58	0:05:13	63.1%	52.4%	10.9%
360000	360000	0:26:41	0:07:24	0:15:11	0:06:38	72.2%	56.4%	10.5%
400000	400000	0:30:11	0:09:02	0:17:15	0:08:06	70.1%	53.0%	10.3%

#### DL trace algorithms

**Fig. 17:**
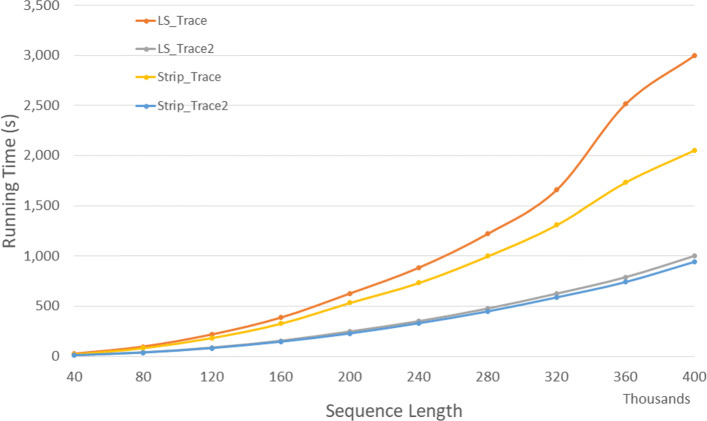
Run time for DL trace algorithms on Xeon24

Figure 17 and Table 15 give the run times of our single-core trace algorithms on our Xeon24 platform. *Strip*_*Trace*2 is the fastest followed by *LS*_*Trace*2,*Strip*_*Trace*, and *LS*_*Trace* (in this order). *Strip*_*Trace*2 reduces run time by up to 9.4*%* relative to *LS*_*Trace*2 and by up to 57.4*%* relative to *Strip*_*Trace*.

**Table 15:** Run time in *hh*:*mm*:*ss* for DL trace algorithms on Xeon24

A	B	LS_Trace	LS_Trace2	Strip_Trace	Strip_Trace2	L vs L2	S vs S2	L2 vs S2
40000	40000	0:00:25	0:00:12	0:00:21	0:00:11	52.7%	49.7%	9.4%
80000	80000	0:01:36	0:00:40	0:01:23	0:00:37	58.5%	55.4%	7.1%
120000	120000	0:03:38	0:01:28	0:03:04	0:01:22	59.5%	55.5%	7.0%
160000	160000	0:06:26	0:02:36	0:05:27	0:02:26	59.5%	55.4%	6.6%
200000	200000	0:10:26	0:04:08	0:08:54	0:03:47	60.3%	57.4%	8.4%
240000	240000	0:14:44	0:05:51	0:12:14	0:05:30	60.3%	55.0%	6.0%
280000	280000	0:20:22	0:07:57	0:16:39	0:07:28	61.0%	55.2%	6.1%
320000	320000	0:27:39	0:10:26	0:21:51	0:09:48	62.3%	55.1%	6.1%
360000	360000	0:41:56	0:13:10	0:28:55	0:12:21	68.6%	57.3%	6.2%
400000	400000	0:50:01	0:16:42	0:34:16	0:15:43	66.6%	54.1%	5.9%

#### Parallel algorithms

**Fig. 18:**
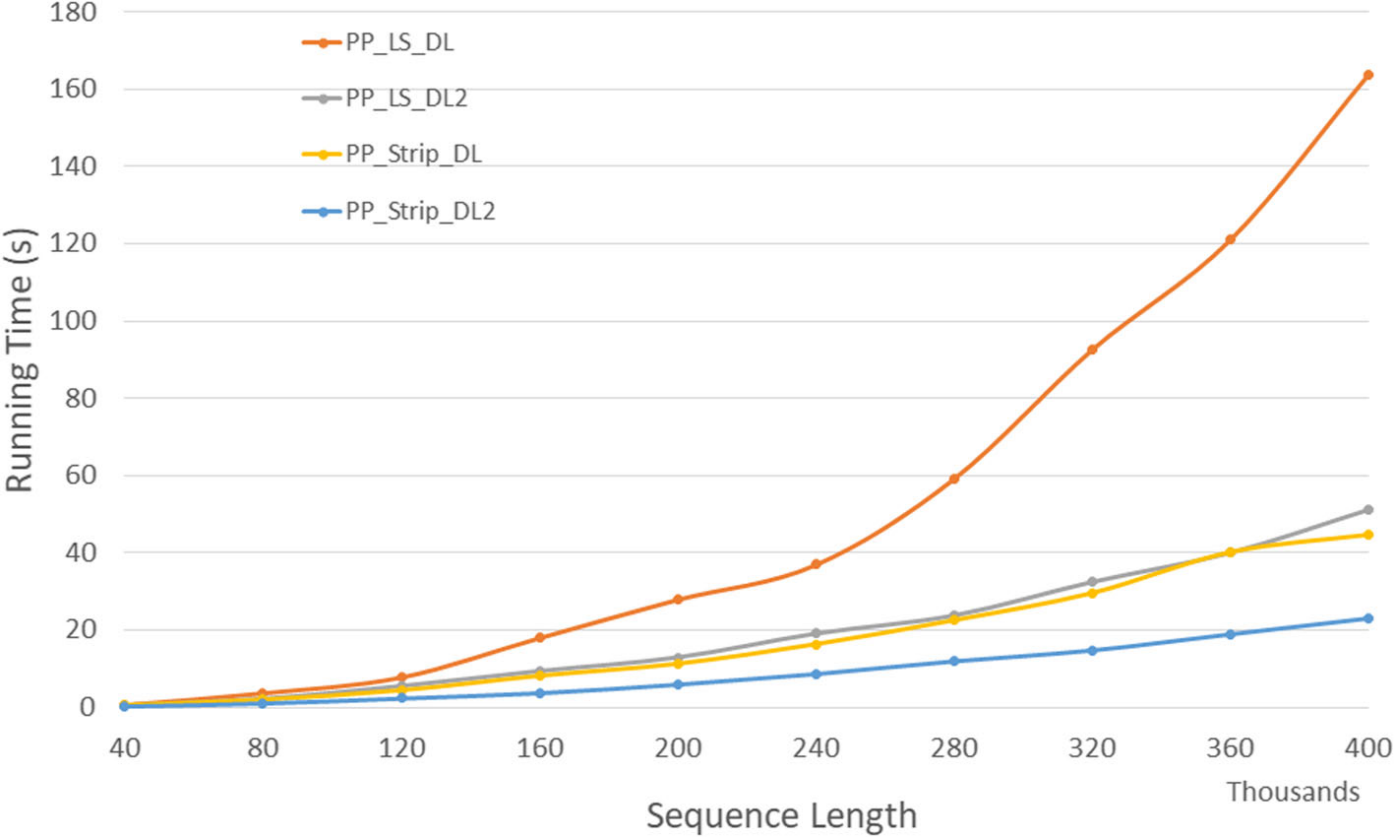
Run time of parallel DL distance algorithms, in seconds, on Xeon24

Figure 18 and Table 16 give the run times for our parallel DL distance algorithms on our Xeon24 platform. *PP*_*LS*_*DL*2 is up to 68.7*%* faster than *PP*_*LS*_*DL* and *PP*_*Strip*_*DL*2 is up to 54.6*%* faster than *PP*_*Strip*_*DL*. Also, *PP*_*LS*_*DL*2 and *PP*_*Strip*_*DL*2 achieves a speedup up to 11.2 and 21.36 compared to the corresponding single core algorithms on a twelve-four core machine, respectively.

**Table 16:** Run time of parallel DL distance algorithms, in *hh*:*mm*:*ss*, on Xeon24

A	B	PP_LS_DL	PP_LS_DL2	PP_Strip_DL	PP_Strip_DL2	L vs L2	S vs S2	L2 vs S2
40000	40000	0:00:01	0:00:01	0:00:01	0:00:00	30.9%	48.6%	41.6%
80000	80000	0:00:04	0:00:02	0:00:02	0:00:01	41.8%	47.2%	51.4%
120000	120000	0:00:08	0:00:06	0:00:04	0:00:02	30.2%	47.4%	57.4%
160000	160000	0:00:18	0:00:09	0:00:08	0:00:04	48.1%	54.6%	60.3%
200000	200000	0:00:28	0:00:13	0:00:11	0:00:06	53.8%	47.6%	54.2%
240000	240000	0:00:37	0:00:19	0:00:16	0:00:09	48.4%	47.3%	54.8%
280000	280000	0:00:59	0:00:24	0:00:23	0:00:12	59.8%	47.2%	50.0%
320000	320000	0:01:33	0:00:32	0:00:30	0:00:15	65.0%	50.3%	54.8%
360000	360000	0:02:01	0:00:40	0:00:40	0:00:19	66.9%	53.1%	53.0%
400000	400000	0:02:44	0:00:51	0:00:45	0:00:23	68.7%	48.7%	55.3%

**Fig. 19:**
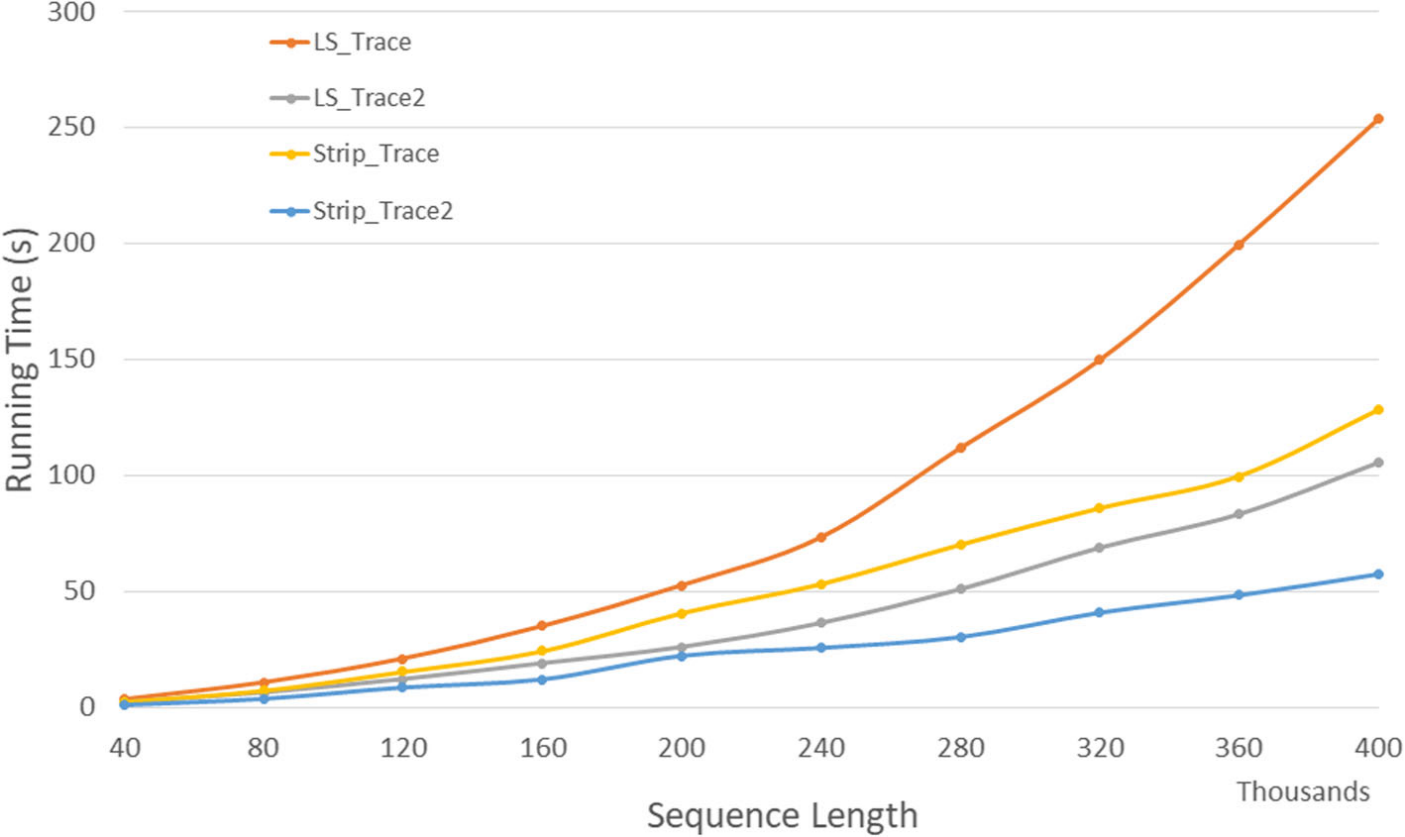
Run time of parallel DL trace algorithms, in seconds, on Xeon24

Figure 19 and Table 17 give the run times for our parallel DL trace algorithms on our Xeon24 platform. *PP*_*LS*_*Trace*2 is up to 58.3*%* faster than *PP*_*LS*_*Trace* and *PP*_*Strip*_*Trace*2 is up to 59.3*%* faster than *PP*_*Strip*_*Trace*. Also, *PP*_*LS*_*Trace*2 and *PP*_*Strip*_*Trace*2 achieves a speedup up to 9.65 and 16.4 compared to the corresponding single-core algorithms, respectively.

**Table 17:** Run time of parallel DL trace algorithms, in *hh*:*mm*:*ss*, on Xeon24

A	B	PP_LS_Trace	PP_LS_Trace2	PP_Strip_Trace	PP_Strip_Trace2	L vs L2	S vs S2	L2 vs S2
40000	40000	0:00:03	0:00:02	0:00:02	0:00:01	38.0%	59.3%	53.3%
80000	80000	0:00:11	0:00:06	0:00:07	0:00:04	40.3%	49.2%	42.5%
120000	120000	0:00:21	0:00:12	0:00:15	0:00:09	42.3%	44.1%	29.2%
160000	160000	0:00:35	0:00:19	0:00:24	0:00:12	45.8%	50.2%	36.5%
200000	200000	0:00:53	0:00:26	0:00:41	0:00:22	50.6%	45.2%	14.2%
240000	240000	0:01:13	0:00:36	0:00:53	0:00:26	50.4%	51.7%	29.4%
280000	280000	0:01:52	0:00:51	0:01:10	0:00:30	54.4%	56.7%	40.4%
320000	320000	0:02:30	0:01:09	0:01:26	0:00:41	54.0%	52.3%	40.6%
360000	360000	0:03:20	0:01:23	0:01:40	0:00:48	58.2%	51.4%	41.9%
400000	400000	0:04:14	0:01:46	0:02:08	0:00:58	58.3%	55.1%	45.5%

## Discussion

We have developed linear space algorithms to compute the DL distance between two strings and also to determine an optimal trace (edit sequence). Besides using less space than the space efficient algorithms of [18], these algorithms are also faster. Significant run-time improvement (relative to known algorithms) was seen for our new algorithms on all three of our experimental platforms.

## Conclusion

On all platforms, the linear-space cache-efficient algorithms *Strip*_*DL*2 and *Strip*_*TRACE*2 were the best-performing single-core algorithms to determine the DL distance and optimal trace, respectively. *Strip*_*DL*2 reduced run time by as much as 56.4*%* relative to the *Strip*_*DL* and *Strip*_*TRACE*2 reduced run time by as much as 57.4*%* relative to the *Strip*_*Trace*. Our multi-core algorithms reduced the run time by up to 59.3*%* compared to the best previously known multi-core algorithms.

The linear space string correction algorithm developed in this paper requires 2*S*≤*I*+*D*≤2*T*, where *S*, *I*, *D* and *T* are, respectively, the cost of a substitution, insertion, deletion and transposition. As noted earlier, this requirement is met by the DL distance as for this metric, *S*=*I*=*D*=*T*=1. When only *I*+*D*≤2*T* is satisfied, the more general algorithm ([18]) that runs in *O*(*mn*) time and uses *O*(*s*∗*min*{*m,n*}+*m*+*n*) space, where *s* is the size of alphabet comprising the strings, may be used.

## Data Availability

Data sharing is not applicable to this article as all test data are randomly generated protein sequences.

## Abbreviations

CPU: Central Processing Unit
DL: Damerau-Levenshtein
DNA: DeoxyriboNucleic acid
LLC: Last level cache
LRU: Least recently used
NCBI: National Center for Biotechnology Information
PDB: Protein Data Bank
RNA: RiboNucleic acid
